# Delving into the Inception of BODIPY Dyes: Paradigms of In Vivo Bioimaging, Chemosensing, and Photodynamic/Photothermal Therapy

**DOI:** 10.3390/ph19010169

**Published:** 2026-01-18

**Authors:** Olivia Basant, Edgardo Lobo, Gyliann Peña, Maged Henary

**Affiliations:** 1 Department of Chemistry, Petit Science Center, Georgia State University, 100 Piedmont Avenue SE, Atlanta, GA 30303, USA; obasant1@student.gsu.edu (O.B.); elobo107@gmail.com (E.L.); gpena6@student.gsu.edu (G.P.); 2 Center for Diagnostics and Therapeutics, Georgia State University, 100 Piedmont Avenue SE, Atlanta, GA 30303, USA

**Keywords:** boron dipyrrin, aza-BODIPY, photosensitizer, Stokes shift, bioconjugate, chemosensor, near-infrared dyes, fluorescent dyes, tumor imaging, theranostics

## Abstract

Boron-dipyrromethene (BODIPY) dyes belong to a class of organoboron compounds that have become ubiquitous for researchers in areas of fluorescence imaging, photodynamic therapy, and optoelectronics. The intrinsic qualities of BODIPY dyes and their meso-modified structural analogs, Aza-BODIPY dyes, have propelled their recent increase in use in biomedical applications. The two scaffolds have high quantum yields, narrow absorption, and emission bandwidths with large Stokes’ shifts, and high photostability and thermal stability. Because their properties are independent of solvent polarity and dye functionality, they can be tuned to promote novel analytical methods, resulting in the adaptation of the physicochemical and spectral properties of the dyes. In this review of BODIPY and Aza-BODIPY scaffolds, we will summarize their spectral properties, synthetic methods of preparation, and applications reported between 2014 and 2025. This review aims to summarize the advances in chemosensing, especially pH sensor development, and the advances in NIR-II window bioimaging probes. We hope that this succinct overview of Aza-BODIPY scaffolds will highlight their untapped potential, elucidating insights that may catalyze novel ideas in the physical organic realm of BODIPY.

## 1. Introduction

During the last few decades, novel dye chemistry has conferred significant advancements in cell imaging modalities using fluorescent probes. However, despite these significant strides in optical imaging, light emitted by fluorescent probes exhibits the tendency to be absorbed by blood cells and proximal cells, resulting in a suppressed contrast and low signal-to-noise ratio [1]; a high signal-to-noise (SNR) ratio is desired to ensure a clear bioimage is generated. A few probes emit around 800 nm, such as 4,4-difluoro-4-bora-3a,4a-diaza-s-indacene (BODIPY), which are dyes that have strong ultraviolet (UV) absorbing molecules that can emit sharp fluorescence peaks with high quantum yields. BODIPY-centered π–π* transitions have been reported to display absorption peaks within the range of 570–640 nm and emission peaks around 615–664 nm with quantum yields of up to 25% [2]. Developing fluorophores with a larger Stokes shift affords a larger window for greater spectral acquisition in quantity of scans with higher resolutions at multiple wavelengths, supporting the propensity of imaging into the NIR-II window (1000–1700 nm) [3]. One differentiating characteristic of BODIPY dyes that distinguishes them from other classes of small molecular organic dyes is the central complexation of two fluorine atoms to a boron atom that is connected to a dipyrromethene ligand; the dipyrromethene ligand is formed from two pyrrole rings connected by a polymethine bridge. The dipyrromethene ligand forms the core scaffold that binds to the boron atom, which is essential to the dye’s fluorescent properties [4]. These components synergize to give BODIPY dyes their photophysical characteristics. The employment of NIR BODIPY dyes seeks to accomplish absorbance and fluorescence in the near-infrared region (NIR). This region encompasses the NIR-I and NIR-II classes, which exist between the 650–900 nm and 900–1700 nm range, respectively, rendering them optimal for biomedical applications [5]. Optical imaging is extensively employed in vivo for research purposes, offering tissue contrast that hinges on the interplay between photons and cellular structures, especially by other classes of dyes, such as donor-π-acceptor fluorophores [6] and squaraine fluorophores [7].

**Figure 1:**
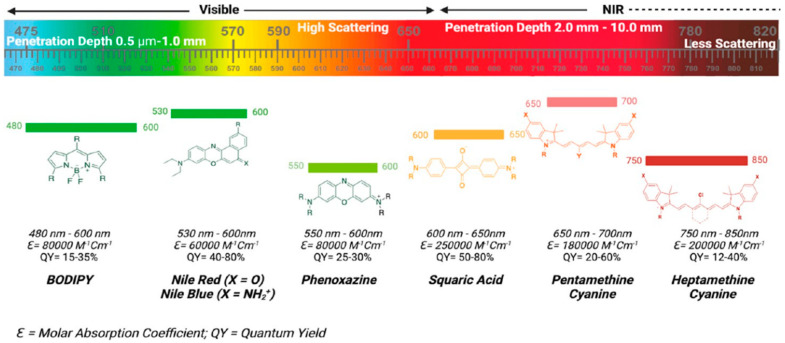
Prominent structures used with numerous classes of NIR fluorophores, with their absorption/emission profiles meeting the candidacy for potential agents for in vivo imaging. This image was reproduced with permission from Shamim et al., Synthesis, Optical Properties and In Vivo Biodistribution Performance of Polymethine Cyanine Fluorophores; published by [American Chemical Society], (2023) [12].

The mechanism by which fluorophores are responsible for causing fluorescence occurs through the absorption of photons in the ground singlet state, leading to vibrational relaxation in the first singlet excited state. As a result, the electron reverts to its ground state, emitting a photon during this relaxation process, known as fluorescence [8]. Fluorescence imaging has become an essential mechanism for monitoring in vivo biological targets due to its non-invasive, real-time imaging abilities and a lack of associated radiation. Fluorescence imaging is a non-invasive technique that allows for the infiltration of animal tissues due to its ability to generate longer wavelengths and low energy with negligible scattering, as well as due to the lack of interference with the innate autofluorescence of biological tissues, making NIR dyes an exemplary contender for bioimaging applications [9,10]. As displayed in Figure 1, the notable classes of NIR-I fluorophores are squaric acid, pentamethine cyanine, and heptamethine cyanine fluorophores. At the same time, the BODIPY core exists at the lowest wavelength and penetration depth among the displayed fluorophore classes, warranting the need for future development of BODIPY probes [11].

**Figure 2:**
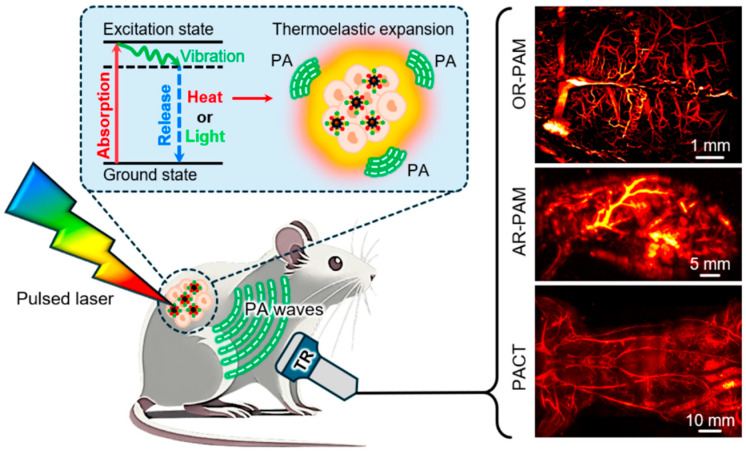
Visual depiction of the principles that govern photoacoustic imaging and its variations. Abbreviations denoted: PA, photoacoustic; TR, ultrasound transducer; OR-PAM, optical resolution photoacoustic microscopy; AR-PAM, acoustic resolution photoacoustic microscopy; PACT, photoacoustic computed tomography. This image was reproduced with permission from Kye et al., Photoacoustic Imaging of pH-Sensitive Optical Sensors in Biological Tissues; published by [MDPI], (2024) [15].

In the last decade, BODIPY dyes have been integral in their employment in two prominent imaging modalities: optoacoustic imaging and fluorescence imaging for photodynamic therapy. Exogenous contrast agents play an integral role in optical imaging due to their highly modifiable structures and ability to improve pre-existing physicochemical and optical properties to constitute bright fluorophores that provide detailed, high-resolution images in image-guided surgery [13]. First, optoacoustic imaging, also known as photoacoustic imaging (PAI), is a non-invasive medical modality that entails laser pulses being directed at biological tissues to induce the expansion of tissues to generate ultrasound waves and is reliant on the absorption of light to elicit a signal response to subsequently propagate ultrasound waves for signal acquisition [14]. As photons propagate through the tissue, light is absorbed by the exogenous contrast agent (dye), and the tissues are locally heated and subjected to expansion. This increase in pressure from the expansion of the tissue results in ultrasound waves, which are detected by an ultrasound transducer to reconstruct an image, as shown in Figure 2 [14].

**Figure 3:**
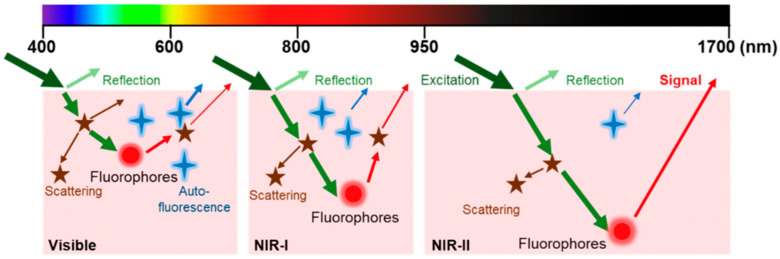
Comparison of fluorescence imaging in different biological regions, such as the visible region (400–700 nm), the NIR-I region (700–900 nm), and the NIR-II region (950–1700 nm). This graphic was reproduced with permission from Zhang et al., Optical and Photoacoustic Imaging In Vivo: Opportunities and Challenges; published by [American Chemical Society], (2023) [17].

In photodynamic therapy, the transition of fluorophores from the excited to the ground state via the absorption of light in the near-infrared (NIR) region is succeeded by the emission of light, known as fluorescence. Absorbed energy, scattered and dissipated as non-radiative energy, assumes the form of thermal heat because of the vibronic relaxations occurring within the excited state of the fluorophore [16]. When this heat dissipation exceeds the rate of absorption, tissue temperatures begin to rise gradually above 40 degrees Celsius, at which cellular tumor cytotoxicity typically occurs. As displayed in Figure 3, in the NIR I and II region, at imaging wavelengths of 700–1700 nm, there is less scattering and absorption of light by hemoglobin and water is relatively low, leading to deeper penetration [17].

Henceforth, this review investigates the current paradigms of BODIPY dyes in their synthetic methodologies, reported applications, and optical properties. The review summarizes the applications of notable BODIPY and Aza-BODIPY dyes synthesized in the last 10 years due to the strides in the development of BODIPY fluorophores that have successfully served as chemosensors for detrimental analytes, such as reactive oxygen species (ROS), and/or simultaneously expanded their clinical potential, promoting selective cancer cell uptake, plasma membrane (PM) targeting, and inducing pyroptosis,—with the ultimate goal of this class of NIR dyes to be leveraged as efficient theranostic agents.

## 2. Synthesis of BODIPY Dyes

**Figure 4:**
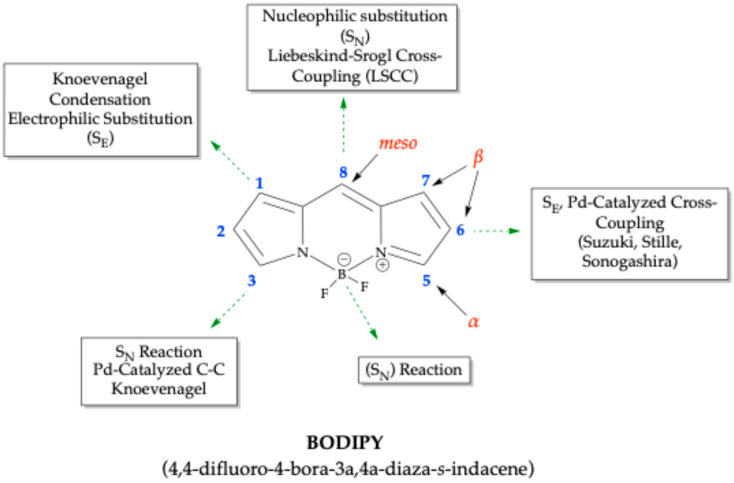
Overlay of approaches used to adopt different moieties into the core of the BODIPY scaffolds and increase the absorption of wavelength (λ_max_).

The first BODIPY dye was discovered in 1968 by Triebs and Kreuzer, and the dyes have since been investigated for their propensity to behave as bimodal imaging agents. Strategies to modify the substituents attached to the core have been directed towards ligand tuning with electron-donating groups and halogenation to promote intersystem crossing [18]. Strategies for synthesizing functional BODIPY fluorophores have encompassed multicomponent reactions (MCRs), metal-catalyzed C-H activation, cycloadditions, and biomolecule-based methods, such as the inclusion of tyramine amino acid units [19,20], as shown in Figure 4. The various strategies that have been adopted to synthesize the NIR BODIPYs aza-BODIPYs include the following: (1) establishment of π-conjugated systems like aryl, vinyl or styryl groups at the 2nd, 3rd, 5th and 6th position of the BODIPY core [21]; (2) replacement of the meso-position carbon with nitrogen atom to form aza-BODIPYs; (3) functionalizing the boron center by replacing fluorine with different atoms or groups of atoms, in place of borylation; (4) further functionalizing at the meso position through meso modifications such as C-C cross couplings; and (5) extension of π-conjugation by fusing (alpha fusion or beta fusion) different aromatic rings to enhance the rigidity in the system due to the supplemented π-conjugation and improve π-π stacking [21].

BODIPY dyes are impervious to most pH and polarity changes within their environment and can withstand physiological conditions [22,23]. By making small modifications to their structures, it is possible to tune their fluorescence characteristics. BODIPY fluorophore dyes feature a chromophore that enables the design of tailor-made molecules for specific biological and technological purposes [24,25]. They have tunable properties that are famous for their chemical versatility and flexible photophysical and electrochemical properties, such as electrochemiluminescence (ECL), that can aid their abilities to be used in chemosensing and serve as labels for bioimaging [19,26]. Last, BODIPY dyes undergo degradation mechanisms in both acidic and basic media and displayed no decomposition after three days in deuterated chloroform (CDCl_3_) or pure chloroform (CH_2_Cl_2_) solutions [27,28].

### General Synthetic Strategies for BODIPY Dyes

Dye chemistry has gained traction in organic chemistry since the first BODIPY dye was discovered in 1968; its popularity allowed for the new development of molecular structures that deck the chromophore’s backbone. Since advanced techniques of synthesizing unique functional fluorophores have been made possible, exploration of chemotypes that were not accessible in the past due to the limitations of conventional approaches is now possible. Classical synthetic methods, such as amide formations, Knoevenagel reactions, and Suzuki couplings [29,30], enable the ability to increase the quantum yield by designing compounds that will dominate in radiative photon emission processes rather than losing energy from rotatable bonds to non-radiative decay pathways, ultimately increasing the molecular brightness of the fluorophores, as displayed in Figure 4.

**Scheme 1:**
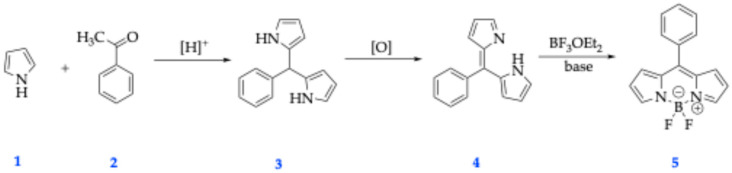
Schematic depiction of the acid-catalyzed condensation of example aromatic aldehydes with pyrrole en route to the formation of BODIPY fluorophores.

There are two commonly distinct synthetic approaches to obtain BODIPY dyes for use in porphyrin research, one of which is the condensation of aldehydes **2** with pyrrole **1** that yields a dipyrromethene as shown in Scheme 1, below [31,32]. To prevent polymerization, these reactions must be carried out in a solvent of pyrrole. For the best yields, dipyrromethenes are used immediately after preparation, as they are unstable in solution and are sensitive to light. Dipyrromethene **3** or dipyrrin **4** can undergo oxidation by DDQ or p-chloranil, which yields dipyrromethene. Then the addition of base to dipyrrin and boron trifluoride etherate leads to the creation of boron difluoride complex **5**. Using this synthetic approach, two equivalents of a substituted pyrrole undergo reaction with one equivalent of an acyl chloride, yielding an unstable dipyrromethene intermediate. This intermediate is then subjected to a strong base like triethylamine (TEA) along with a boron source to generate the BODIPY dye.

**Scheme 2:**
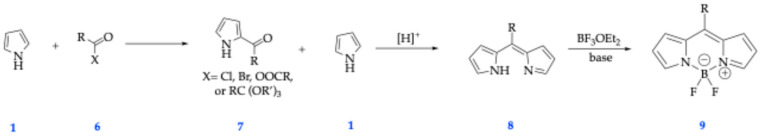
Schematic depiction of the acid-catalyzed condensation example reaction of acylpyrrole with an additional pyrrole en route to the formation of BODIPY fluorophores.

However, a different synthetic route uses condensation with another pyrrole and an acylium **6**. An acylium is an acyl group, such as a carboxylic acid, that can be derivatized into other functional groups. The acylium may be an acid chloride, anhydride, or orthoester [33,34]. The intermediate acylpyrrole **7** is not usually isolated and is committed to further reaction under acidic conditions with excess pyrrole **1** to yield dipyrrin **8**; this technique has been used to develop meso-substituted 3-pyrrolyl BODIPY fluorophores as precursor substrates to prepare a series of BODIPY-metal dipyrrin conjugates using palladium (Pd), ruthenium (Ru), and rhenium (Re) [34]. After the condensation, the addition of excess base and boron trifluoride etherate can also yield fluorescent BODIPY dyes (**9**) as shown in Scheme 2.

## 3. Synthesis of Aza-BODIPY Dyes

Currently, there are three prominent routes for the synthesis of Aza-BODIPY dyes: (1) O’Shea’s, (2) Carreira’s, and (3) Lukyanets’ methods, which were all discovered in the early 2000s, specifically between 2002 and 2008, and are displayed in Table 1. O’Shea et al. (2002) [35] developed a novel method to create symmetrical and asymmetrical aza-BODIPY dyes via cyclization of 1,3-diaryl-4-nitrobutan-1-one and subsequently reported that the replacement of the methine carbon at the meso-position with a nitrogen results in a red shift without lowering the extinction coefficients and fluorescence intensities of the non-meso-substituted parent dyes. Similarly, Shi et.al [33] Wang et.al [36] employed O’Shea’s synthesis to develop small molecules for bioimaging application and for conformationally restricted ring fused BODIPYs, respectively. Carriera et al. (2005) [37] constructed symmetrical and asymmetrical aza-BODIPY structures from 2,4-diaryl-pyrroles and NaNO_2_. Finally, Lukyanets et al. (2008) [38] leveraged the ubiquity of Grignard chemistry to prepare aza-BODIPYs from a reaction between phthalonitrile and aryl Grignard reagents [36]. In addition to the three previously mentioned synthetic routes, Aza-BODIPY units have also been employed as linker and acceptor groups, due to the conjugated and photochemically stable BODIPY core to be leveraged as a scaffold for D-π-A fluorophores [6,36]. The inherent electron-withdrawing ability of the BF_2_ group enables the BODIPY structure to be leveraged as an acceptor unit for several donor groups to be attached to it.

### 3.1. O’Shea’s Route

**Scheme 3:**
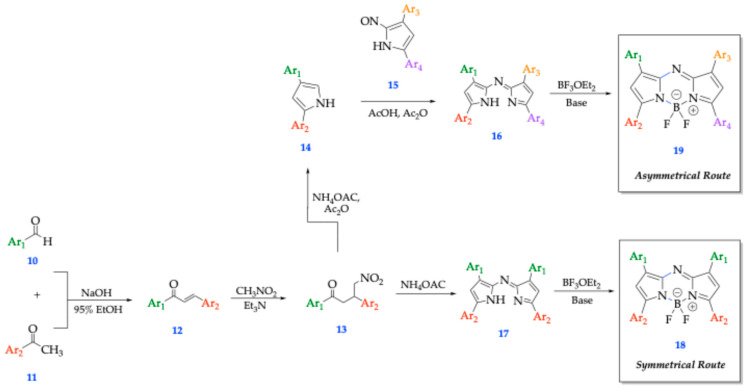
Symmetrical and asymmetrical Aza-BODIPY fluorophore synthetic route through O’Shea’s method [36].

As presented in Scheme 3, the chalcone can be used to form a nitro chalcone or a 2,4-disubstituted pyrrole, which then undergoes intramolecular cyclization to form the UV-active fluorophore and establish the conjugated polymethine backbone chain of the fluorophore. In chalcone **12**, aryl ring 1 (Ar_1_) denotes the substituent contributed from the benzaldehyde **10**, and aryl ring 2 (Ar_2_) denotes the substituent contributed from the acetophenone **11**. The mechanistic overview of O’ Shea’s route involves 3 steps: (1) Michael Addition, (2) intramolecular cyclization, and (3) boron complexation. Chalcone **12** is subjected to Michael Addition under reflux with diethylamine and nitromethane to add a nitro (-NO_2_) group to the β (beta) position of the diketone, forming a nitro-substituted intermediate— aptly named “nitro chalcone” **13**.

When forming symmetrical Aza-BODIPYs, the two units of the nitro chalcone **13** react in a 2:1 ratio with excess ammonium acetate in the presence of a high-boiling solvent, such as butanol (BuOH) or acetic anhydride (Ac_2_O), where the nitro group is reduced to a pyrrolic amine, in the presence of ammonium acetate (NH_4_OAc). Typically, the pyrrolic amine **14** is not isolated and used in situ as the reaction is allowed to proceed to completion. Herein, the 1st pyrrolic amine unit is reduced to yield compound **15** and reacts with another equivalent of the nitro chalcone diketone to form a transient diamino ketone intermediate. This ephemeral structure is not isolated and undergoes intramolecular cyclization, leading to the formation of the Aza-dipyrromethene core **17**. When forming asymmetrical Aza-BODIPYs, two unique nitro chalcones bearing four different aryl (Ar_X_) substituents are reacted in a 2:1 ratio to form two units of pyrrolic amines, which will react in the same fashion as the symmetrical route, to form the asymmetrical aza-dipyrromethene core **16**. Finally, in both the symmetrical and asymmetrical case, the aza-dipyrromethene intermediate is deprotonated in the presence of a base such as triethylamine (TEA) or *N*, *N*-diisopropylamine (Hünig’s base) where the nucleophilic attack by the Lewis acid BF_3_ from the source of boron trifluoride yields the final BF_2_ complexed fluorophores **18** and **19** as outlined in Scheme 3.

### 3.2. Carriera’s Route

**Scheme 4:**
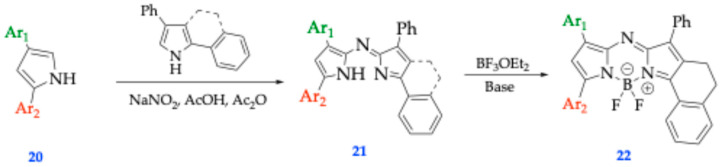
Asymmetrical Aza-BODIPY fluorophore synthetic route through Carriera’s method of Schiff base formation [36].

In Carriera’s Route, the synthesis of the Aza-BODIPY fluorophores is characterized by the direct cyclization of 2,4-diaryl pyrroles **20** to form the BODIPY core, or the BODIPY core is formed through Schiff base formation from a lactam (i.e., phthalimide) and a heteroaromatic amine to form a Schiff base intermediate **21**, characterized by a C=N double bond [36]. The Schiff base undergoes intramolecular cyclization under acidic conditions, forming a dihydro-azadipyrromethene intermediate. The dihydro-azadipyrromethene is treated with boron trifluoride diethyl etherate in the presence of a base such as triethylamine to form the final fluorophore **22** as outlined in Scheme 4.

### 3.3. Lukyanets’ Route

**Scheme 5:**
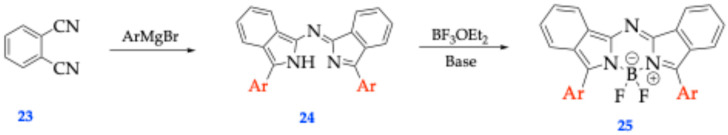
Symmetrical Aza-BODIPY fluorophore synthetic route through Lukyanets’ method of using Grignard reagents [36,38].

In 2008, the third method for synthesizing Aza-BODIPY fluorophores was pioneered by Lukyanets et al. (2008) [38], who developed this novel route to establish a phthalocyanine analog Aza-BODIPY. Through the reaction of phthalonitrile (1,2-dicyanobenzene), as seen in Scheme 5, compound **23** reacts with aryl magnesium bromide in dry benzene at room temperature for 1 h [38]. First, a nucleophilic addition of the Grignard reagent, aryl magnesium bromide, adds to the nitrile carbon of the phthalonitrile, resulting in an unstable, short-lived intermediate diimine (Ar-C=N-Ar’), which undergoes intramolecular condensation via an acid-catalyzed or thermally driven reaction to lead to the aza-dipyrromethene, as shown in structure **24**, containing the central nitrogen in the six-membered ring, flanked by two pyrrole-like aromatic rings. Finally, the synthesis is completed by the chelation of boron to the two-basic site pyrrolic nitrogen atoms, resulting in the planar, rigid aza-BODIPY dye, as seen in structure **25**. The structure of compound **25** was revealed through single crystal X-ray analysis and revealed that the fluorophore adopts a near C_2v_ symmetric conformation, in which the isoindole substituents are fixed in a coplanar manner, and the coordination geometry around the boron atom is similar to Aza-BODIPY dyes synthesized through O’Shea’s and Carriera’s method, as evidenced by B–N distances of 1.573 Å and 1.579 Å and by two fluorine atoms with B–F distances of 1.372 Å and 1.380 Å, respectively [38,39].

### 3.4. Strategies to Achieve Bathochromic Shift

**Figure 5:**
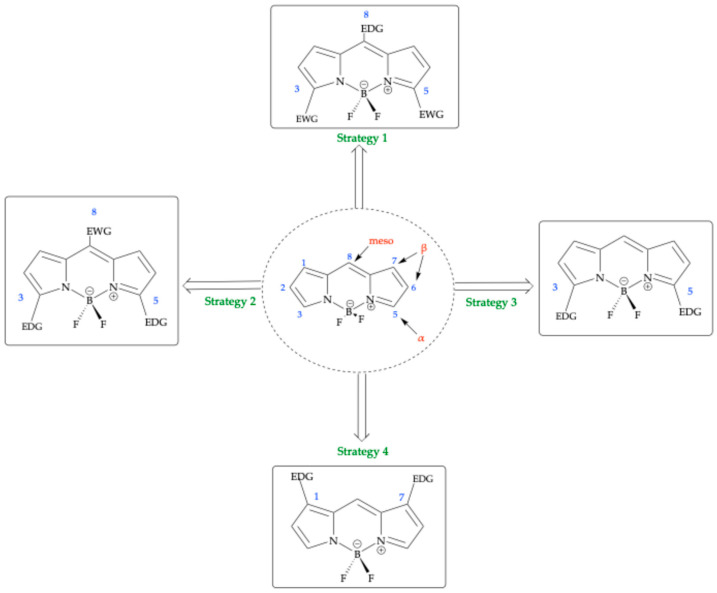
Four types of synthetic modifications to favor a further push into the NIR (red shift) [31].

Prominent strategies to red shift the wavelength are highlighted in Figure 5, which include (1) extending the conjugation of the dye system and (2) increasing the rigidity of the BODIPY core. The absorption and emission of a compound are directly influenced by electronic conjugation. Therefore, the most common design strategy for shifting BODIPY molecules towards red wavelengths is extending conjugation. Increasing the extent of the conjugation reduces the energy gap between the frontier orbitals—the highest occupied molecular orbital (HOMO) and the lowest unoccupied molecular orbital (LUMO)—resulting in more energy being conserved to be converted into longer absorbance wavelengths. There are various approaches to designing an expanded delocalized π system, including extending π conjugation and increasing rigidity. Incorporating aryl, styryl, or similar functionality at the 1, 3, 5, and 7 positions of the BODIPY core can effectively extend π conjugation. For simplicity in synthesis, conjugation is frequently extended only at the 3- and 5-positions of the BODIPY core, demonstrating the spacer effects of donor-π spacer-acceptor sensitizers [40]. Expanding conjugation involves adding more π electrons to a delocalized system, thereby increasing the number of molecular orbitals and consequently narrowing the HOMO-LUMO gap. Similarly, by virtue of increasing the system’s planarity via increased conjugation, restricting the degree of rotatable bonds increases the rigidity of the BODIPY core. Unrestricted rotation along the bonds connecting the BODIPY and an attached π spacer can result in misalignment of the π orbitals, thereby diminishing the impact of π conjugation. Additionally, bulky alkyl groups that are sterically constrained can be added to the core to enhance steric hindrance, thereby fixing or restraining the degree of rotation inherent to the π spacer (or linker). Torsion angles can be reduced by either fusing aromatic rings or by incorporating five-membered heterocycles instead of aryl functionality [22,37].

### 3.5. Synthetic Modifications Favoring Bathochromic Shift

The creation of a narrow band-gap chromophore is possible by incorporating intramolecular charge transfer (ICT) properties into the molecule. An ICT molecule undergoes a transfer of electron density of an electron (rich) donor group (EDG) through a π-conjugated linker to an electron (poor) withdrawing group (EWG) [31]. The π linker, depending on the type, acts as an insulator or conductor and undergoes the charge transfer process. The introduction of charge transfer is an effective way to modulate the excited state by altering the emission geometry. This procedure stabilizes the lowest unoccupied molecular orbital (LUMO), thereby influencing the emission wavelength [26,30]. Figure 5 entails various ways to synthetically modify the BODIPY core to achieve a bathochromic shift. Strategy 1 entails placing an EDG group at the 8 position (aza) and two EWGs on positions 3 and 5 on the core, resulting in large MO coefficients in both the LUMO and HOMO. Strategy 2 entails placing an EWG group at the 8 position (aza) and two EDGs on positions 3 and 5 on the core results, which should narrow the HOMO-LUMO gap, making for a red-shifted dye. Strategy 3 entails incorporating an EDG at 3 and 5 positions, which should raise the HOMO energy level, causing a longer wavelength. In 2009, the first reported effect of chalcogens bathochromically shifting BODIPY absorbance maximums was reported, described as the periodic trend that descends a group of chalcogens (from O, Se, S, and Te) employed at 3 and 5 positions increases longer wavelength absorption [41]. Furthermore, recent advances in functionalization at the 3 and 5 positions to shift the excitation/emission bands were created by targeting the 3 and 5 positions to install phenyl boronic acid (PBA) as a means of also tethering glycan domains to the streptavidin monoclonal antibody, to also address the bottleneck of versatility among the BODIPY dyes [42]. Likewise, it is a known trend that the higher the strength of the electron-donating species (EDG), the greater the energy of the highest occupied molecular orbital (HOMO) [26,30,31]. Finally, strategy 4 entails an EDG being placed at the 1 and 7 positions because there are no nodal planes in the HOMO near these positions. Generally, it can be inferred that electron-donating groups (EDGs) exert a stabilizing influence on the highest occupied molecular orbital (HOMO) by elevating its energy when appropriately attached to the BODIPY core. Here, there are nodes; a region of no electronic density exists. Thus, the more nodes present, the higher the energy of the system [31].

### 3.6. Novel Boron Modifications to the BODIPY Difluoride Core

**Scheme 6:**
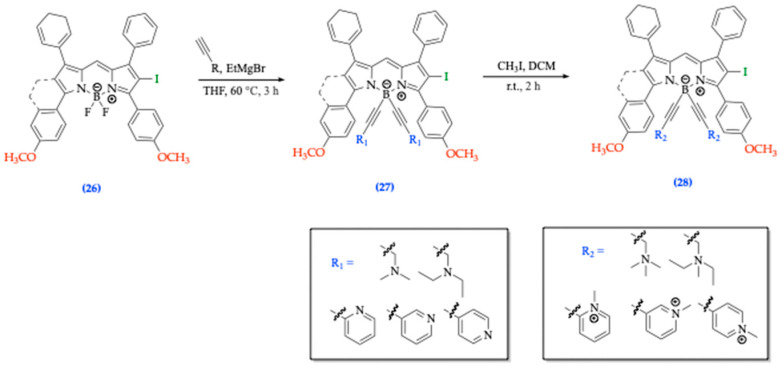
Synthesis of boron-modified aza-BODIPY PSs and respective reagents and conditions: (1) Alkyne-based reagents (10 equiv.), ethylmagnesium bromide (10 equiv.), tetrahydrofuran, 60 ◦C, 3 h; yield: 30–87%. (2) Methyl iodide (10 equiv.), dichloromethane, ambient temperature, 2 h; yield: 48–69%. This figure was adapted from Hu et al., 2025, Novel boron-modified aza-BODIPY photosensitizers for low-dose light-dependent anti-cancer photodynamic therapy; published by [Elsevier], (2025) [43].

**Figure 6:**
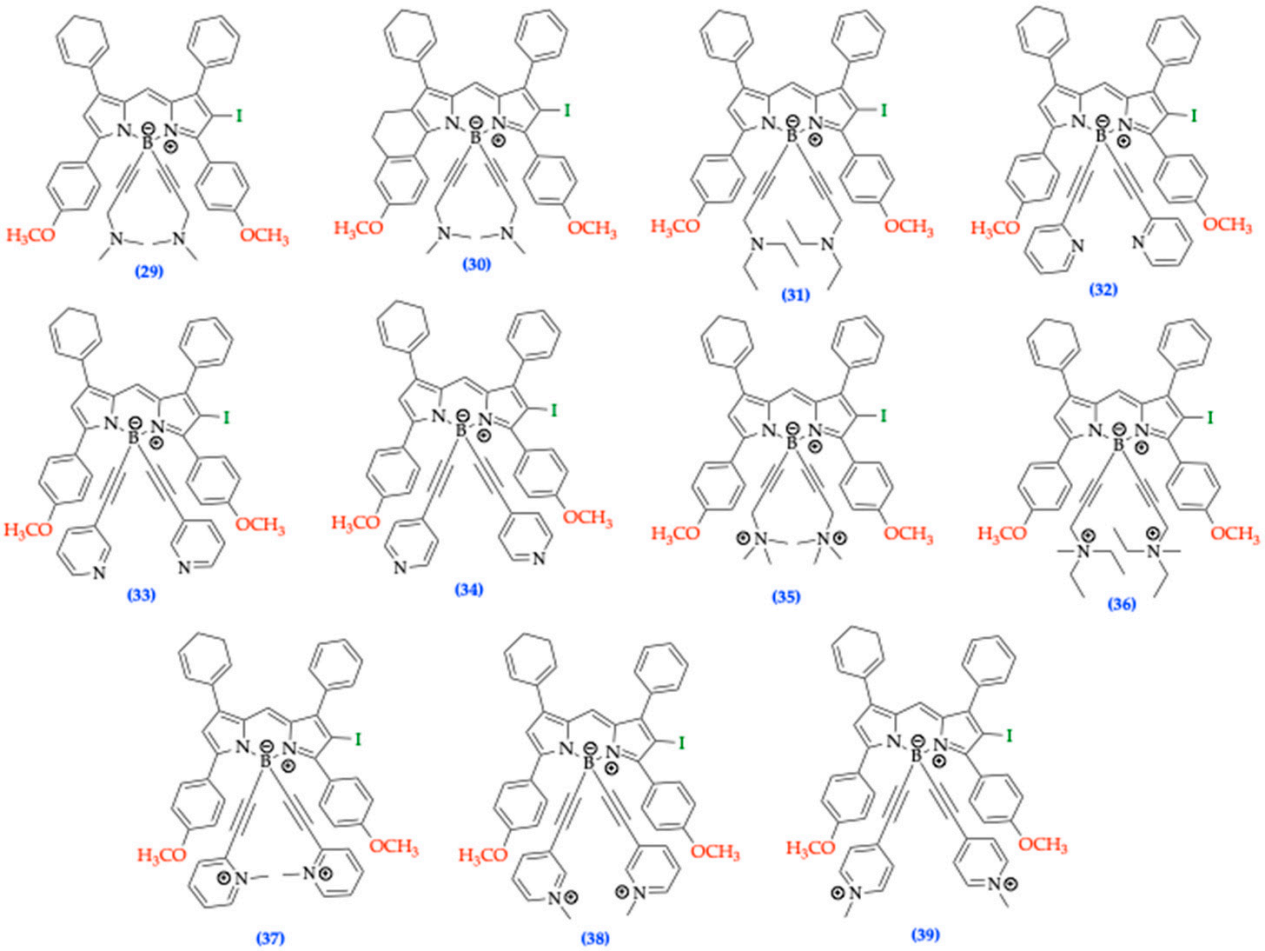
Structural depictions of alkynyl and alkylated alkynyl Aza-BODIPY derivatives probed for improved photodynamic efficacy [43].

Recently, an emerging strategy to expand the selectivity and therapeutic efficacy of Aza-BODIPY fluorophores for photodynamic therapy includes changing the difluoride unit of the fluorophore into cyano (-CN) and alkynyl units. These substitutions create a predisposition for further derivatives. For instance, Hu et al., (2025) demonstrated how the installation of alkynyl units promoted low-dose light-dependent anti-cancer photodynamic therapy [43]. A widely recognized limitation of the Aza-BODIPY dye class is that the large, planar conjugated structure and high lipophilicity create a tendency for Aza-BODIPY photosensitizers to aggregate in aqueous solutions, which leads to diminished photosensitizing activity. Hu et al. (2025) created structures that installed the rigidified alkynyl chains on the boron atom to improve their amphilicity without altering the spectroscopic properties; when amphilicity improves, this phenomenon enhances the singlet oxygen quantum yield [43]. The alkynyl derivatives were designed to suppress intermolecular π-π stacking, thereby reducing susceptibility to aggregation and ultimately, fluorescence quenching, while retaining photosensitive activity. Scheme 6 displays generic BODIPY compounds **26**–**28**, which display the varying “R” substituents corresponding to the new alkynyl derivatives **29**–**39**, as shown in Figure 6. The synthesis begins with the introduction of alkyne-based reagents (10 equiv.) and Grignard reaction, ethyl magnesium bromide (10 equiv.) in a 1:1 ratio using tetrahydrofuran (THF) as a solvent, followed by alkylation with methyl iodide (10 equiv.) in dichloromethane at ambient, room temperature to afford the respective alkynyl units in yields 48–69% [43].

**Figure 7:**
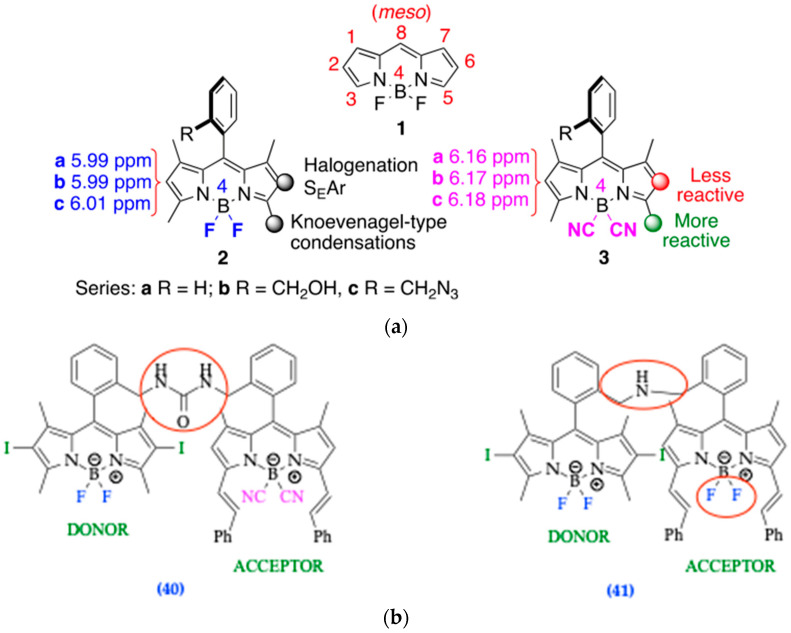
(**a**) Side-by-side comparison of the 4,4′-difluoro-BODIPY and the 4,4′-dicyano-BODIPY cores. Note the more deshielded pyrrolic protons occurring at a higher chemical shift, due to the stronger electron-withdrawing substituent, cyano, installed. (**b**) Depiction of fluorophores **40** and **41** that were obtained via the site-selective modifications displayed in (**a**). These figures were reproduced with permission from Ventura et al. 2023, 4,4′-Dicyano- versus 4,4′-Difluoro-BODIPYs in Chemoselective Postfunctionalization Reactions: Synthetic Advantages and Applications; published by [American Chemical Society], (2023) [44].

Moreover, Ventura et al. (2023) explored the chemoselective post-functionalization reactions to confer synthetic advantages by modifying BF_2_ BODIPYs to contain a dicyano (CN)_2_ substitution, which displayed enhanced reactivity in Knoevenagel condensations with aldehydes [44]. As displayed in Figure 7, Ventura et al. (2023) [44] displayed the observed reactivity between the 4,4′-difluoro-BODIPY and the 4,4′-dicyano-BODIPY, showing the pyrrolic protons corresponding to positions 2 and 6 within the difluoro-BODIPY core. Of the numerous derivatives Ventura et al. (2023) [44] synthesized to advance the development of BODIPY dimers and trimers for light-harvesting applications, the authors developed BODIPY dimer fluorophores **40** and **41** to display balanced fluorescence emission and singlet oxygen generation. After previously optimizing structures through the iodination of urea-bridged versus amino-bridged chromophores, the respective dimers **40** and **41** were synthesized, where fluorophore **41** displayed a slightly lower fluorescence efficiency in chloroform at 34% but possessed a higher level of singlet oxygen generation at 36% [44], indicating that the intersystem crossing population driven by heavy-atom iodine was virtually eliminated.

## 4. BODIPY Dyes as Fluorescent Sensors

**Figure 8:**
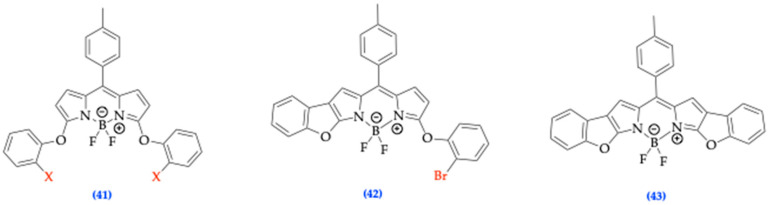
Structural depictions of benzofuran BODIPY derivatives probed for spectral properties [46].

The works of Bardon et al. (2018) [45] developed a variety of adaptable reactive systems, which are readily substitutable. One prominent application of these systems is their utilization in fabricating fluorescent sensors. Given the high sensitivity of fluorescence response measurement, this method can be used for detecting biologically significant substrates. Significantly, the potential to incorporate nitrogen nucleophiles at the 3 and 5 positions could be intriguing, as substitution at these positions directly impacts the spectral characteristics of the dye [45]. Moreover, the spectroscopic properties of transition metal-catalyzed benzofuran formation BODIPYs **41**, **42**, and **43**, as outlined in Figure 8, demonstrate one of the earliest reported instances of the rigidification of the BODIPY structure via annulation, an efficient strategy to confer higher molar extinction coefficients and fluorescence quantum yield (ϕf) values [46]. The progressive enhancement in the planarity of the chromophore within the series **41** → **42** → **43** explains the rising bathochromic shifts observed in both λ_abs_ (max) and λ_em_ (max). By increasing the stiffness of systems **42** and **43** through benzofuran formation and by increasing the planarity of the chromophore, the degrees of vibrational freedom decrease.

**Figure 9:**
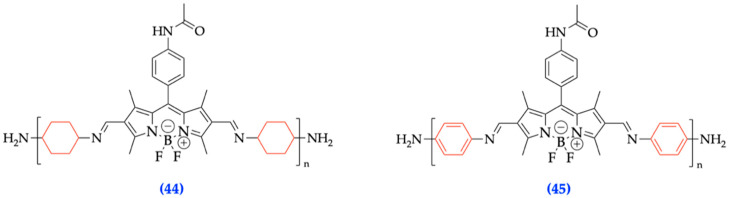
Fluorophores **44** and **45**, which have been used as fluorescent sensors in the detection of explosives and picric acid [47].

Recent advances in BODIPYs as fluorescent sensors have been achieved through combining spectral properties and synthetic reactive systems from 2014 to 2025. One new advent has been the employment of BODIPYs as explosive sensors for the tracing of picric acid (PA), hexahydro-1,3,5-trinitro-1,3,5-triazine (RDX), and peroxides through a fluorescence turn-on mechanism. Compounds **44** and **45**, as shown in Figure 9, were developed to detect picric acid and showed fluorescence emission at 473 nm, but fluorescence was quenched in the presence of picric acid. Their respective limit of detection (LOD) was at 0.038 and 0.017 µM, respectively [47]. Moreover, Sadikogullari et al. 2023 [48] demonstrated the quenching responses of Aza-BODIPYs in response to the interaction with nitroaromatics (NACs), using fluorescence titrations. Fluorescence studies were carried out using four explosive media (TNP, TNT, and DNT), where it was observed that the presence of *π* delocalization in nitro-bearing compounds led the aza-BODIPY fluorophores to become quenched in as little as 7–11 min, with TNP becoming detectable in as little as 80 s and taking fully 7 min to quench fluorescence [48].

**Figure 10:**
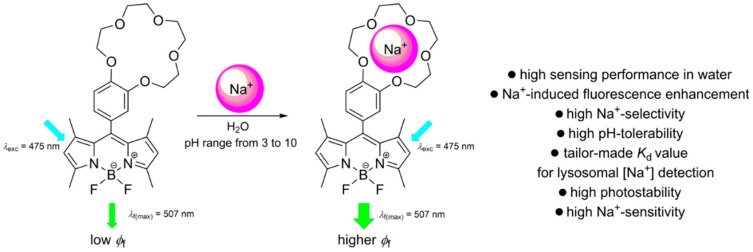
Schematic depicting the mechanism of action of compound **47**, which quenches fluorescence through protonation because the excited fluorophore experiences contact with an atom or molecule that can facilitate non-radiative transitions to the ground state. This figure was reproduced with permission from Sprenger et al., 2022, BODIPY-Equipped Benzo-Crown-Ethers as Fluorescent Sensors for pH Independent Detection of Sodium and Potassium Ions; published by [Wiley-VCH GmbH], (2023) [49].

Another advance in the development of BODIPY fluorescent sensors is the concept of a “fluoroionophore” that is highly selective for sodium cations (Na^+^). BODIPY-equipped benzo-crown-ethers were employed as fluorescent sensors for the independent detection of sodium and potassium ions [49], equipped with different-sized macrocycles to facilitate binding to and subsequent fluorometric detection of biological Na^+^ and K^+^ levels, as depicted in Figure 10. BODIPY ionophore **49** displayed significant fluorescent enhancement (FE) within the pH range from 3 to 5, due to the protonation of the anilino nitrogen donor. As a result, the protonation of the anilino nitrogen blocks the fluorescence quenching process and triggers the FE. Compared to the BODIPY ionophores **46**, **47**, and **48**, which exhibited a highly stable fluorescence signal across a pH range from 3.5 to 9.5, they were subjected to fluorescence titration experiments with alkali metal ions (Na^+^, K^+^, and Li^+^) at different pH values. Compound **47** displayed a higher affinity for Na^+,^ and compound **48** formed a more stable complex with K^+^ than with Na^+^, as indicated by their dissociation constants displayed in Table 2.

**Figure 11:**
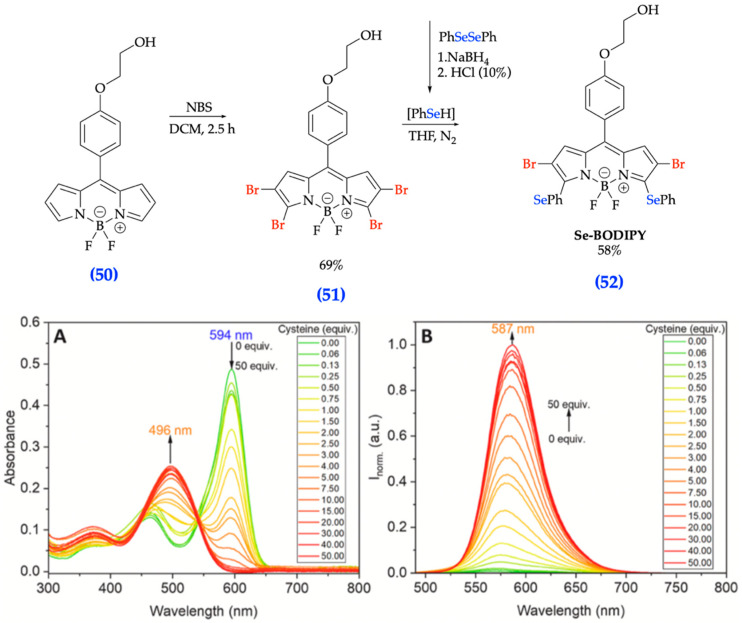
(**A**) Absorption and (**B**) emission spectra profiles of Se-BODIPY (7.6 μmol/L) in THF/H_2_O 50% *v*/*v* pH 7.0, upon addition of increasing concentration of Cys (0–380 μmol/L; 0–50 equiv.), after 30 min of reaction. λ_ex_ = 480 nm, slits 3.0/3.0 nm. These figures were reproduced with permission from Cugnasca et al., Seleno-BODIPY as a fluorescent sensor for differential and highly selective detection of Cysteine and Glutathione for bioimaging in HeLa cells; published by [Elsevier] (2025) [50].

**Figure 12:**
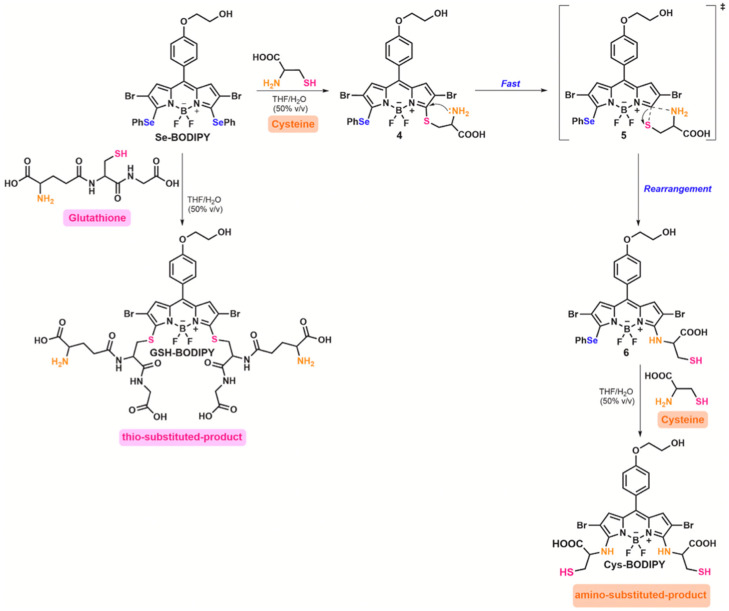
Proposed reaction mechanism of Se-BODIPY, compound **52**, with Cys and GSH. This image was reproduced with permission from Cugnasca et al., Seleno-BODIPY as a fluorescent sensor for differential and highly selective detection of Cysteine and Glutathione for bioimaging in HeLa cells; published by [Elsevier] (2025) [50].

Finally, a Seleno-BODIPY fluorescent sensor for differential and highly selective detection of bio thiols, cysteine (Cys), and glutathione (GSH) was developed by Cugnasca et al. (2025) [50]. Compound **52** displayed colorimetric and turn-on fluorometric responses to Cys and GSH, exhibiting low detection limits of 61.2 nM and 1.66 µM, respectively, as shown in Figure 11. Compound **52** was screened against 26 analytes including amino acids, reactive sulfur species (RSS), and anions, where it was possible to verify selectivity and expressive responses for bio thiols like Cys, *N*-acetylcysteine (NAC), GSH, Homocysteine (Hcy), and sodium sulfide (Na_2_S) via absorbance of whether they exhibited a color change from blue to orange (Cys) and pink (NAC and GSH) [50]. After obtaining emission spectra, λ_ex_ = 488 nm was employed to detect Cys and λ_ex_ = 553 nm was employed to detect GSH. Through a titration with increasing amounts of Cys (0–50 equiv.), a decrease at λ_max_ = 594 nm and an increase at λ_max_ = 496 nm in a radiometric colorimetric response were detected, with the presence of an isosbestic point, as shown in Figure 11, indicating that the conversion of Se-BODIPY in another product (**Cys-BODIPY**) was observed, as shown in Figure 12.

## 5. Optical Properties of Selected Compounds

### 5.1. Two-Photon Absorption

**Figure 13:**
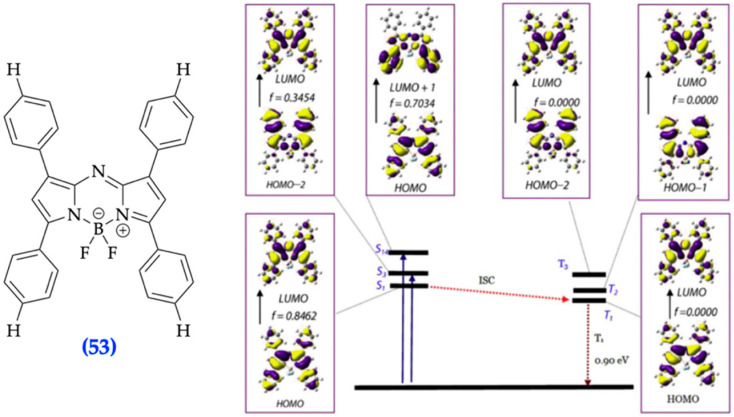
Electronic density map showing the frontier molecular orbitals (highest occupied and lowest occupied) of compound **38**. Computed and analyzed at the B3LYP/6-31G(d)/LanL2DZ level with Gaussian modeling software. This figure was reproduced with permission from Karatay et al., The effect of heavy atoms on two-photon absorption properties and intersystem crossing mechanism in aza-boron-dipyrromethene compounds; published by [Elsevier] (2015) [48].

**Figure 14:**
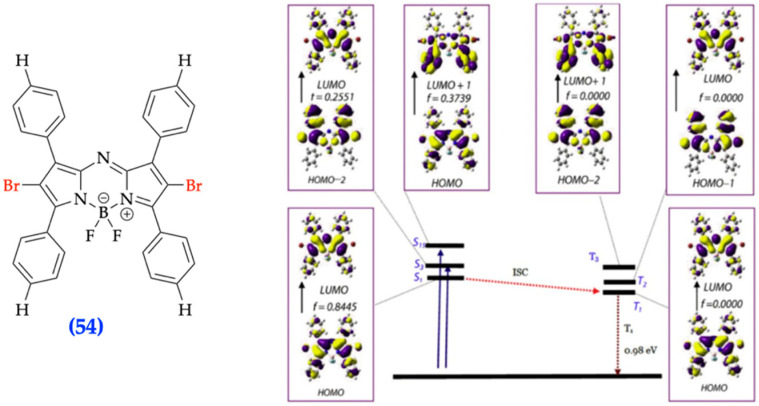
Electronic density map showing the frontier molecular orbitals (highest occupied and lowest occupied) of compound **39**. Computed and analyzed at the B3LYP/6-31G(d)/LanL2DZ level with Gaussian modeling software. This figure was reproduced with permission from Karatay et al., The effect of heavy atoms on two-photon absorption properties and intersystem crossing mechanism in aza-boron-dipyrromethene compounds; published by [Elsevier] (2015) [51].

Karatay et al. (2015) [51] synthesized new Aza-BODIPY fluorophores containing bromine atoms at various positions to enhance the triplet state populations as a means of improving two-photon absorption properties for two-photon photodynamic therapy; the researchers studied the effects of the photoinduced electron transfer mechanism because of the introduction of the heavy-atom, bromine, using ultrafast pump-probe spectroscopy experiments and density-functional theory (DFT) calculations. Ultrafast pump-probe experimental results explained the observed fluorescence quenching by portraying evidence of the intersystem crossing (ISC) mechanism, where the lifetime of the signals shows great differences, whether including Br atoms at the 2 and 6 positions of the aza-BODIPY core or not [51]. To further investigate why compound **54**, with bromines added to the peripheral positions of the BODIPY core, exhibited stronger intersystem crossing (lower fluorescence) than compound **53**, DFT studies were conducted by optimizing the ground state geometries of the compounds, as shown in Figure 13 and Figure 14. Compared to the measured energy value from the first singlet excited state to the first triplet state, it is shown in Figure 14 that compound **54** has a higher energy value of 0.98 eV, compared to compound **38**, than at 0.90 eV for compound **53**, as shown in Figure 13. The DFT studies were imperative to explaining this interaction, as the DFT calculations supported the preliminary results that revealed the singlet and triplet energy levels become closer and more concomitant with each other when the Bromine (Br) atoms are at the 2 and 6 positions of the aza-BODIPY core. The authors implore that their experimental and theoretical investigations, through the introduction of Br onto the peripheral positions of aza-BODIPY compounds, do not affect the triplet state populations [51].

### 5.2. Aggregation-Induced Emission

**Scheme 7:**
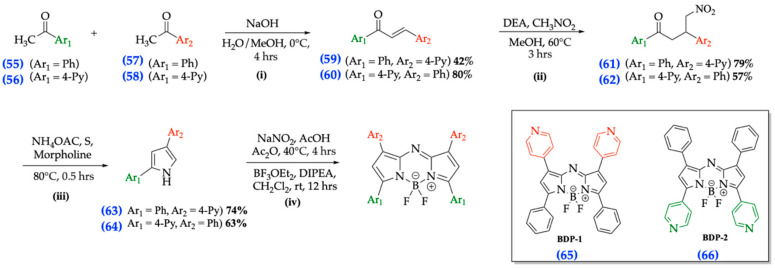
General scheme displaying stepwise synthetic procedure and structures of newly synthesized BDP-1 (**65**) and BDP-2 (**66**) [52].

Given that aggregation of molecules confers a facile way to selectively tune and rationally modify the optical profiles of dyes, a significant challenge still exists in how to control the molecular organization of dye monomers to adopt certain aggregate formations to maximize the efficiency of PDT. Herein, Liu et al. (2023) devised a novel supramolecular strategy to formulate nano-photosensitizers from heavy-atom-free Aza-BODIPYs (BDP-1 and BDP-2), shown as compounds **65** and **66**, as shown in Scheme 7, bearing a pyridine unit, on the benzaldehyde and acetophenone sides of the aza-BODIPY, respectively, via O’Shea’s synthetic route [52]. The general synthetic route for compounds 50 and **51** is displayed in Scheme 7. To further support this tenet, these photosensitizers (PSs) readily form aggregates predominantly in aqueous media because of synergistic π–π stacking and hydrophobic interactions. BDP-1 (**65**) and BDP-2 (**66**) were encapsulated in Pluronic F127 (F127) nanoparticles to serve as an amphiphilic copolymer from nanoparticles, termed BDP-1 NPs and BDP-2 NPs, respectively. However, these niche aggregate formations especially hinder the abundance of reactive oxygen species (ROSs) formation due to the innate self-quenching tendency of the excited state, often resulting in limited clinical utility. It was reported that BDP-1 NPs formed J-aggregates, whereas BDP-2 NPs formed H-aggregates.

**Figure 15:**
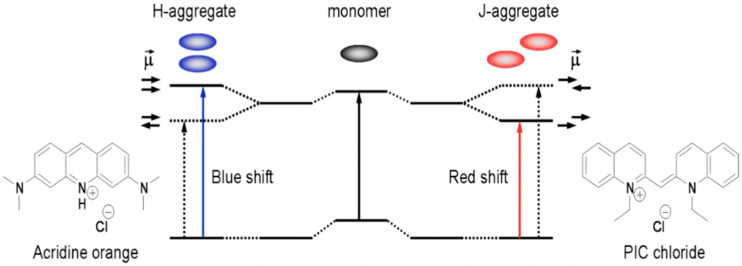
Energy diagrams portray examples of H- and J-aggregates in examples of dyes forming these aggregates, respectively. This figure was reproduced with permission from Klymchenko et al., Emerging field of self-assembled fluorescent organic dye nanoparticles; published by [Springer], (2016) [53].

J-aggregates are aggregates that arise and form because of the transition dipole moments of individual dye monomers undergoing a “slip-stack” alignment, which manifests as an optical tendency to red-shift absorption maxima [24,25], as shown in Figure 15. Furthermore, the HOMO and LUMO frontier orbitals for dyes forming J-aggregates can be segregated efficiently due to the electron transfer to lower split energy levels, conferring an advantageous point to boost the quantum yield (ΦΔ) by decreasing the frontier orbital band gap (ΔEST). Conversely, in H-aggregates, the molecular alignments result from the equilibrium generated from the strong overlapping “face-to-face” contacts. Thereby, by deduction, perfectly aligned H-aggregates are nonfluorescent, as their lowest energy level is forbidden. All in all, it is of keen interest within the arena of dye chemistry to exploit the formation of H-type aggregates of fluorophores, which may emanate energy in other nonradiative forms.

**Figure 16:**
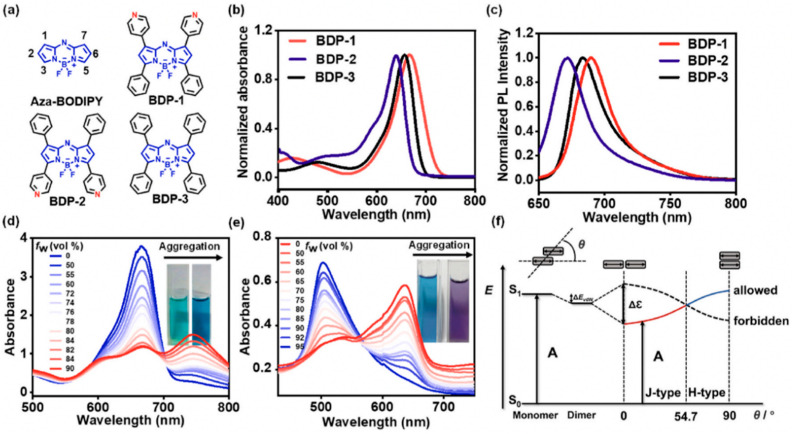
(**a**) Chemical structures BDP-1, BDP-2, and BDP-3; (**b**) normalized absorbance; (**c**) normalized fluorescence intensity; (**d**) BDP-1 and (**e**) BDP-2 in THF/water mixtures with different water fractions (*f_w_*); (**f**) schematic illustration for energy diagram of aggregated dimers. This figure was reproduced with permission from Liu et al., J- and H-aggregates of heavy-atom-free Aza-BODIPY dyes with high ^1^O_2_ generation efficiency and photodynamic therapy potential; published by [Elsevier], (2023) [52].

Moreover, through the introduction of pyridine rings into O’Shea’s synthetic routes of compounds **65** and **66**, it was discovered that their absorption maxima were contingent upon the nature of the substituents installed at the 3 and 5 and 1 and 7 positions of the dipyrrin core. As depicted in Figure 16b, the effect of shifting from the phenyl aryl ring (BDP-3) to the pyridyl (BDP-1) at the 1 and 7 positions resulted in an 11 nm redshift in the pyridyl (BDP-1) optical profile, whereas there was an 18 nm blueshift in the phenyl (BDP-3) optical profile with the 3 and 5 substituents. Likewise, the fluorescence properties of BDP-1 (**65**) and BDP-2 (**66**) were investigated, and they exhibited a similar pattern to that displayed in the absorption spectra, as shown in Figure 16c. These results demonstrated the optical benefit of employing the higher conjugation effect of switching one of the phenyl heterocycles with a pyridyl group in the 1 and 7 positions of the fluorophore core. As a result, the aggregation trends of BDP-1, BDP-2, and BDP-3 were further evaluated by employing a mixed solvent system of water and THF [52,53].

### 5.3. Spectroscopic Properties of Dicationic and Dianionic BODIPY Dyes

**Figure 17:**
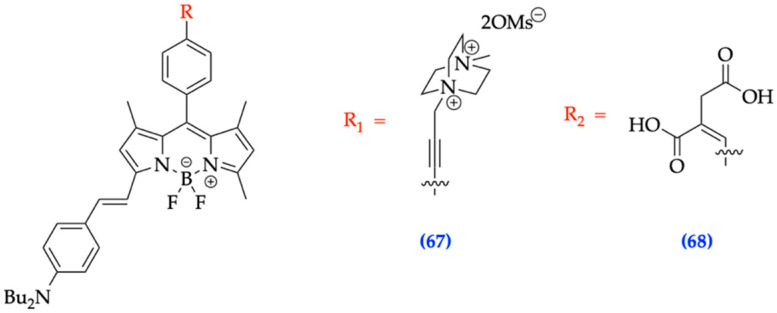
Newly designed BODIPY amphiphilic derivatives **67** and **68** with dibutylaminophenylene vinylene group attached to the 3 position to extend conjugation of chromophore [45].

Bardon et al. (2018) investigated the synthesis of two water-soluble BODIPY dyes, compounds **67** and **68**, as shown in Figure 17, bearing dicationic or dianionic groups, which aided in imparting water solubility and preventing the translocation of the dye through plasma membranes, facilitating their use in aqueous media and minimizing the cytotoxicity issues commonly encountered when organic solvents are used to dissolve and/or load the dyes for bioanalysis and bioimaging applications [45,53]. To prepare photostable, NIR fluorescent water-soluble membrane probes, compounds **67** and **68**, as shown in Table 3, were strategically prepared with combinations of dications and solubilizing agents. Compound **67** employed the use of the triazabicyclo [2.2.2] octane (DABCO) moiety due to its high hydrophilicity across the biological pH range, whereas compound **68** employed the installation of itaconic acid for solubilizing purposes. The parent BODIPY core possessed butyl groups on the amino phenylene unit as a measure of balancing the oleophilic nature of the core. The synthetic strategy conferred the ability of compounds **67** and **68** to be able to partially enter into micelles or membranes while the hydrophilic end allows the compound to be anchored outside of the membrane to interact with the extracellular environment [45]. Our lab, and the previous efforts of Choi et al. (2014) aided in the development of targeted zwitterionic near-infrared fluorophores that exhibited low background, by balancing the total net surface charge distribution of the fluorophore to be 0; this was accomplished by balancing the sulfonate and quaternary ammonium salt substituents to improve tumor targetability [54,55], which is a common modality governing the scope of amphiphilic BODIPY fluorophore synthesis.

## 6. Biomedical Applications

Aza-BODIPY fluorophores find applications in C-H activation reactions, organic solar voltaic cells [10,18], anti-cancer photodynamic and photothermal activity [15,16,17], bioimaging [16,19], and chemosensors [47,49]. Moreover, Aza-BODIPY dyes possess a similar BODIPY core structure except that the nitrogen replaces the carbon atom at the *meso* position. These dyes demonstrate high NIR extinction coefficients and high quantum yields. Aza-BODIPY dyes may be manipulated with hydrophilic groups to facilitate the compound to be leveraged in aqueous environments—an ode to its propensity for in vivo applications. A few examples that can improve solubility and still retain the fluorescence properties include adding quaternary ammonium, sulfonate, or oligo-ethylene glycol moieties to the BODIPY core. The modifications of the aza-BODIPY dye to make it water-soluble allow for the applications of chemosensors’ fluorescence labeling and imaging, pH indicators, and photodynamic therapy [35,56,57].

### 6.1. Metal-Catalyzed C-H Activation Reactions

**Scheme 8:**
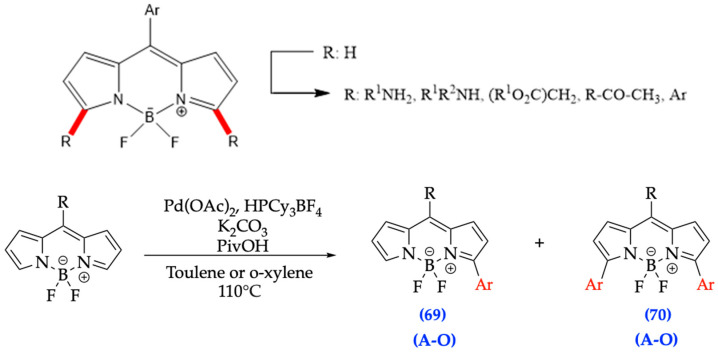
Scheme of C-H arylation of *meso*-substituted BODIPY with different bromoarenes [58].

Metal-catalyzed couplings, such as the Suzuki–Miyaura reactions, are the most common approach to preparing biaryl compounds because there is a need for two functionalized substrates, which include boronic acid and aryl halide. However, a few limitations continue to restrict the availability of substituted boronic acid derivatives. As a result, the limited availability of substituted boronic acid by-products may be overcome with C-H activation processes that directly connect aryl halides to (hetero) arenes by metal-promoted activation of a C-H bond in the latter compound [58,59,60]. Henceforth, Verbelen et al. (2012) [58] developed a general method for preparing brightly fluorescent 3,5-arylated BODIPY dyes with red-shifted electronic spectra, by utilizing C-H functionalization to avoid the cumbersome synthesis of substituted pyrroles to form the BODIPY core. This work was integral to advancing the development of functionalized BODIPY fluorophores for multicomponent reactions from 2014 to 2025. Scheme 8 displays the synthesis of diaryl BODIPY fluorophores **69** and **70**, whereas Table 4 outlines the scope of the direct C-H arylation presented in Scheme 8. Table 4 presents the various C-H arylations and their comparisons of traditional synthesis, denoted superscript “a”, to install different aryl rings, compared to the microwave irradiated protocols, denoted with superscript “b.” As evidenced by selected examples for installing phenyl ring substituents at reaction times of 24 and 28 h in entries 1 and 9, the utility of microwave irradiation to accelerate the reaction is shown in entries 13 and 15. Another example is shown in the installation of 3-thienyl, which improved the reaction time from 27 h to 3 h in entries 4 and 14, respectively.

**Scheme 9:**
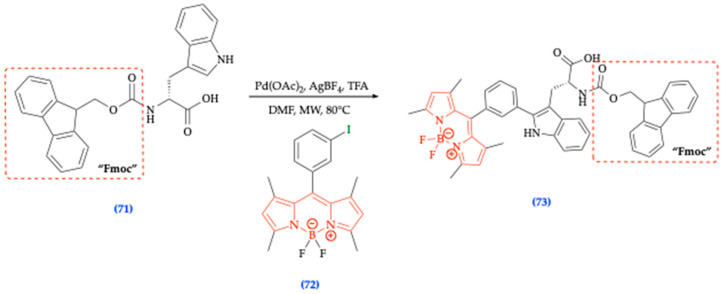
Synthetic scheme of Trp-BODIPY fluorogenic amino acid **73** [61].

**Figure 18:**
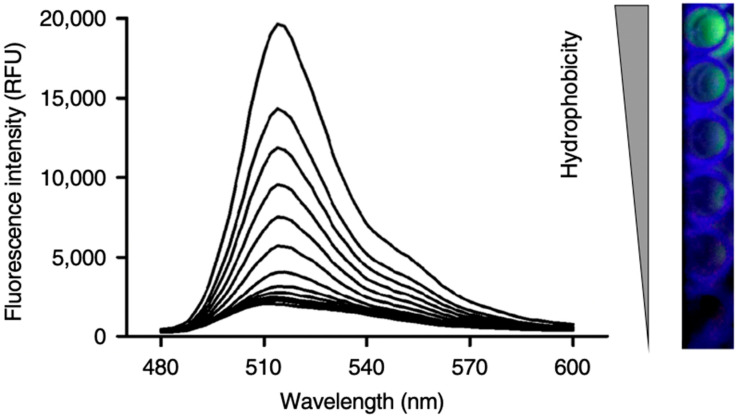
Spectral properties of compound **73** at 10 μM were obtained after incubation with phosphatidylcholine (PC) and cholesterol (7:1) liposome suspensions in PBS. This figure was reproduced with permission from Mendive-Tapia et al., Spacer-free BODIPY fluorogens in antimicrobial peptides for direct imaging of fungal infection in human tissue; published by [Springer Nature], (2016) [61].

Synthesis of fluorogenic tryptophan (Trp) serves as a critical building block for the preparation of peptide-based fluorophores [61,62]. These peptide-based fluorophores are synthesized in single-step reactions that result in good yields. These reactions are coupled using meta iodophenyl-substituted BODIPY **72** and Fmoc-Trp-OH in the company of Pd (OAc)_2_ by microwave irradiation, as shown in compound **73**, as shown in Scheme 9. Subsequently, the Trp–BODIPY amino acid was combined with antimicrobial peptides to label the fungal pathogen *Aspergillus fumigatus* in ex vivo human tissue [61]. Notably, their activity and selectivity were not deterred by peptides, and this created several chances for the development of novel peptide-based imaging probes. Compound **73**’s interaction with the phospholipid bilayer membranes was studied concerning its co-culture with human lung epithelial cells, where it was established that the use of tryptophan as a fluorogenic surrogate caused increased hydrogen bonding to peptides within the lipid bilayer, promoting the increased fluorescence intensity, as shown in Figure 18. The use of the Fmoc protection was due to mitigating degradation during the solid-phase peptide synthesis [58].

### 6.2. Functionalized BODIPY Fluorophores for Multicomponent Reactions (MCRs)

**Figure 19:**
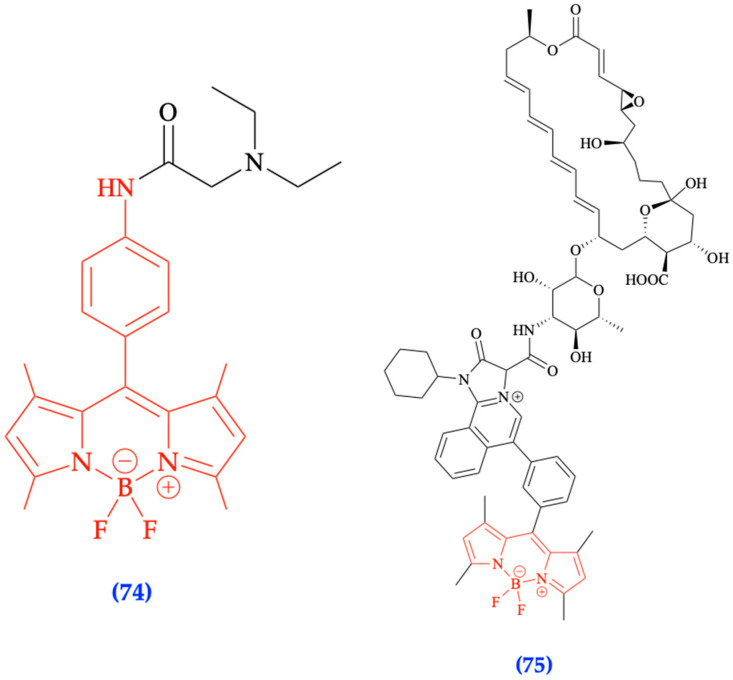
Functional fluorophore obtained by MCR, PhagoGreen, as a pH-sensitive BODIPY fluorophore for in vivo imaging of phagocytic macrophages [62]. MCR’s synthetic approach has been used for isoquinoline-substituted BODIPY structures with labels of bioactive amines and fluorescent analogs [52,57].

Multicomponent reactions (MCRs) are precursors colored to differentiate the multicomponent nature of the syntheses. MCRs are derived from their convergent character, modular features, and access to novel chemotypes, such as those based on the FRET principles, to create water-soluble chitosan BODIPY derivatives that present good photostability, as successfully achieved by Zhou et al. (2018) [63]. Moliner de Fabio and colleagues explored the applicability of Ugi 4-CRs, which is a four-component reaction to functionalize fluorophores for optical imaging [62]. Biological analysis has acknowledged PhagoGreen, **74**, as a pH-sensitive fluorophore for imaging phagocytic macrophages in vivo as well as green fluorescent mesoionic BODIPY **75** conjugated to natamycin for live fungal cell imaging, shown in Figure 19. Antimycotic activation is seen through the mesoionic BODIPY compounds [62].

### 6.3. Activatable Photosensitizer Design Considerations

Activatable photosensitizers (aPS) are imaging probes for their nonspecific phototoxicity outside activation or target sites and serve as a molecular activation that differentiates between target cells and healthy cells. Photodynamic therapy (PDT) is a minimally invasive treatment that eliminates target cells in the presence of oxygen by irradiating a photosensitizer with light, producing highly reactive singlet oxygen [64]. Singlet oxygen then attacks cellular targets, which damage cells, resulting in the shutdown of vascular cells and activation of the immune response. When a molecule produces a chemical change in another molecule through a photochemical process, this is known as a photosensitizer. Photosensitizers are used in reactions involving polymer chemistry; examples are photo-polymerization, photo-crosslinking, and photo-degradation.

**Figure 20:**
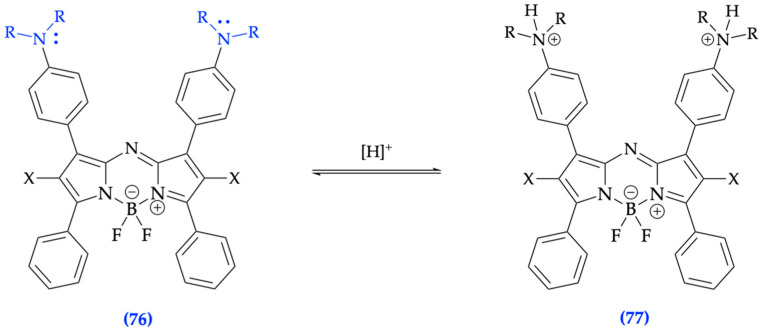
The pH-activatable photosensitizer is based on electron transfer: compounds **76** and **77** [60,61].

PDT has many advantages over traditional therapies. Some worth mentioning are that they are non-invasive, very selective, and capable of giving recurring doses without building resistance or going above total doses [64]. PDTs have small to no scarring, fast healing, an outpatient setting, and no associated side effects, unlike radiotherapy, where patients develop resistance to the dose limitations. Photosensitizers are not only for the therapeutic production of singlet oxygen. They are bright fluorophores and can emit in the NIR part of the spectrum, which is very useful for in vivo imaging. Another way to modulate activation is by using controllable quenching instead of making the photosensitizer singlet oxygen through solvents or pH. Correctly, quenching through photoinduced electron transfer (PET) can be used to control a PS. In pH-activated photosensitizers, they have been shown to successfully kill cells through attaching photoinduced electron transfer-based quenchers that have a specific pKa; they are active in the protonated form, like iodinated BODIPY derivative, such as seen by the unprotonated amino fluorophore **76**, and the protonated amino fluorophore **77**, where fluorescence quenching occurs through the PET process, shown in Figure 20.

**Figure 21:**
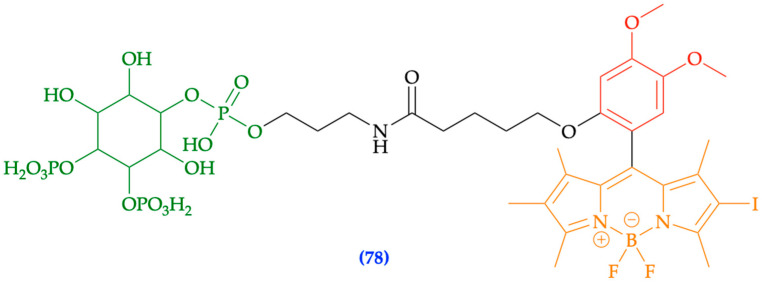
The hydrophobically activatable photosensitizer with protein targeting moiety for chromophore-assisted light inactivation in fluorophore **78** [65].

The next approach mentioned was developed using photoinduced electron transfer quenchers, which are activated in hydrophobic solvents that have low dielectric constants [65]. As shown in Figure 21, compound **78** is an iodinated BODIPY photosensitizer, serving dually as a photoinduced electron transfer (PET) quencher and as a protein-targeting ligand to navigate a desired photosensitizer to the Inositol 1,4,5-trisphosphate (IP3) receptors within the endoplasmic reticulum of eukaryotic cells to mediate the release of Ca^2+^. The photoinduced electron transfer quencher turns out to be wasteful upon binding to the hydrophobic pockets of cellular proteins. To display this phenomenon, iodinated BODIPY photosensitizer (orange) was attached to a photoinduced electron transfer quencher (blue) whose quenching ability was contingent upon solvent hydrophobicity. This led to the inositol 1,4,5-triphosphate ligand (green) to be able to direct the photosensitizer to its protein target, where it was activated by binding in the hydrophobic pocket. This result is indicative of the fluorophore **78**’s ability to be able to be activated to specifically site-selectively damage the desired protein through singlet oxygen generation [65], revealing the inactivation mechanism of certain proteins within live cells.

### 6.4. Biocompatible Aza-BODIPY Biotin Conjugates and Nanoparticles for PDT

**Scheme 10:**
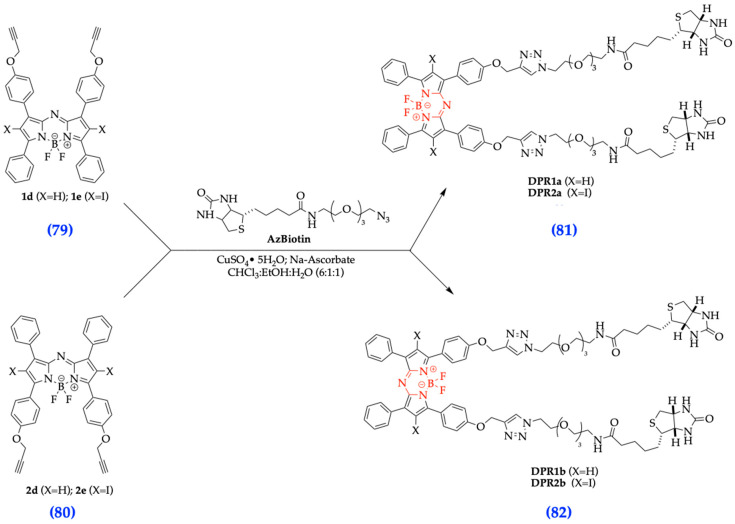
Synthetic pathway developed to form novel fluorophores **66** and **67** with terminal alkyne and copper catalysis to click with the AzBiotin conjugate [66].

Dutta et al. (2023) synthesized the first novel biotin-conjugated Aza-BODIPY dye to be used for PDT, incorporating a three-component framework comprising (1) Aza-BODIPY, (2) PEG-3, and (3) biotin alongside an iodinated BODIPY core to promote intersystem crossing as way to diversify methods to increase fluorescence [66], compared to previous methods used to quench fluorescence through nitrile substitutions [67]. When compared against DPR2a and DPR2b cell lines, the aza-biotin conjugate was found to exhibit significant photocytotoxicity when activated by light, even though it is neither cytotoxic nor toxic in the dark, as compared to Photofrin, the currently most widely used commercial photosensitizer [66,68,69]. As shown in Scheme 10, using click chemistry, fluorophore derivatives **79** and **80** were joined to the azide-containing AzBiotin unit through 1,3-dipolar cycloaddition to yield fluorophore derivatives **81** and **82**. In total, four new biotin-based Aza-BODIPY fluorophores were developed and studied spectroscopically.

As shown in Table 5, the fluorophore DPR1b, represented by fluorophore scaffold **82**, exhibited the highest absorption and emission maxima, as well as the highest fluorescence quantum yield. However, a triplet quantum yield, or the proportion of singlet oxygen yield, could be used to quantify photodynamic activity. In comparison, the fluorophore DPR2b, also shown as fluorophore scaffold **82**, exhibited the highest potential therapeutic capacity, boasting the highest triplet and singlet oxygen generation quantum yields.

### 6.5. Development of NIR-II Aza-BODIPY Fluorophores

**Scheme 11:**
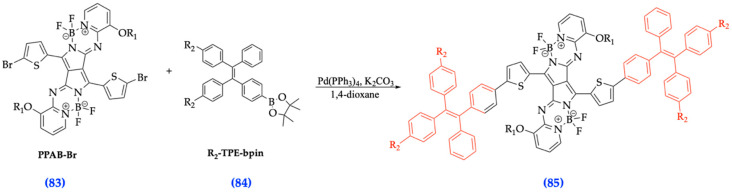
Newly developed tetraphenylethylene (TPE) derivative scaffold that has erred into the NIR-II region (beyond 1200 nm) [70].

**Figure 22:**
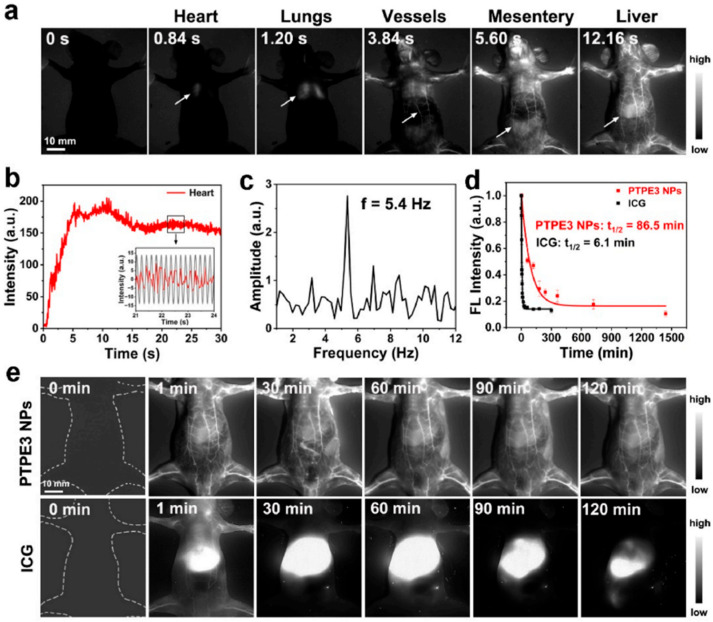
Still-shot images captured during real-life vasculature imaging of vasculature dysfunction processes. (**a**) Images of NIR-II fluorescence seen from the ventral view of the murine model after injecting PTPE3 nanoparticles (exposure time = 30 ms); (**b**) Fluorescence intensity of the heart region within the first 30 s after injection; (**c**) Fourier Transform of the data displayed in frame b; (**d**) Fluorescence intensity of blood samples collected at different time points post-injection of PTPE3 NPs and standard control ICG. (**e**) In vivo NIR-II FL imaging of Balb/c nude mice at different time post-injection of PTPE NPs and ICG. Error bars, mean ± s.d. (*n* = 5/group). This figure was reproduced with permission from Bian et al., Pyrrolopyrrole aza-BODIPY-based NIR-II fluorophores for in vivo dynamic vascular dysfunction visualization of vascular-targeted photodynamic therapy; published by [Elsevier], (2023) [70].

Bian et al. 2023 [70] developed pyrrolopyrrole aza-BODIPY-based NIR-II fluorophores with donor-acceptor-donor (D-A-D) structures by using tetraphenylethylene (TPE) derivatives as electron donors (D) and pyrrolopyrrole aza-BODIPYs (PPABs) as electron acceptors (A). As shown in Scheme 11, the three phenyl groups in the TPE derivatives acted as molecular rotors to endow the fluorophores with aggregation-induced emission (AIE) [63,70,71], properties that could be retained when assembled into amphiphilic polymer nanoparticles (PTPE_3_ NPs). PTPE_3_ fluorophores displayed high brightness (ϵ_max_ Φf > 1000 nm ≈ 180.05 M^−1^ cm^−1^) after encapsulation with amphiphilic polymers and provided long fluorescence emission extending beyond 1300 nm. In Figure 22, it is shown how compound **85** was distributed along the entire circulatory system, with primary accumulation in the mesentery and liver, but Figure 22d shows the nanoparticle-encapsulated fluorophore **85** with a half-life of 86.5 min, which is much longer than the reported half-life of FDA approved ICG, at 6.1 min, indicating high spatial-temporal resolution and prolonged systemic circulation time, which establishes a promising fluorophore for real-time vasculature monitoring of thrombosis formation, vessel occlusion, hemorrhage, and vasculature collapse in vivo [70].

### 6.6. Self-Assembly of Polymeric BODIPY Micelles for Fluorescence Imaging

**Figure 23:**
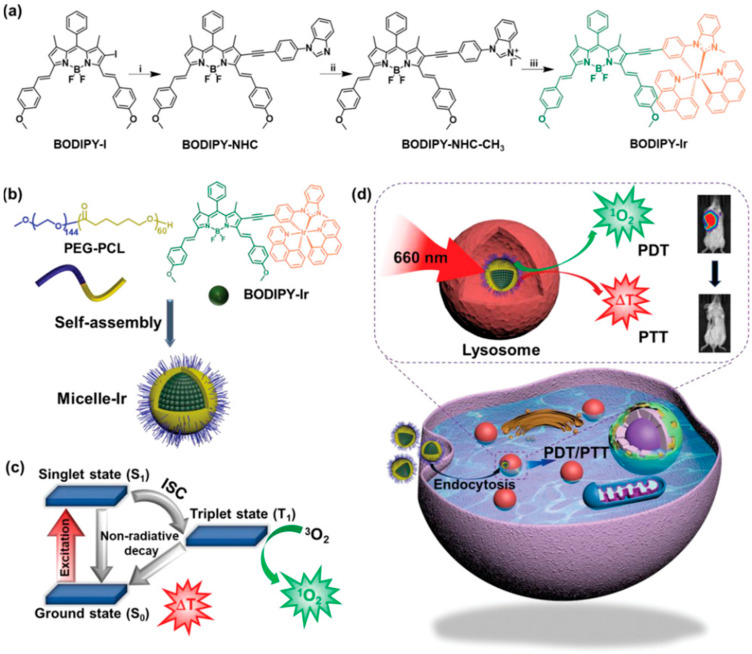
Left to Right: (**a**) Synthetic pathway for obtained iridium liganded fluorophore (**b**) Visual depiction of encapsulation of BODIPY-Ir into micelles for constructing Micelle-Ir; (**c**) Jablonski diagram depicting the photophysical processes leading to singlet oxygen (^1^O_2_) generation and photothermal effect (ΔT); (**d**) Intracellular PDT/PTT effects. This figure was reproduced with permission from Liu et al., Highly Efficient Far-Red/NIR-Absorbing Neutral Ir (III) Complex Micelles for Potent Photodynamic/Photothermal Therapy; published by [Wiley-VCH GmbH], (2021) [72].

To demonstrate the utility of BODIPY as potential NIR photosensitizers (PS) for photodynamic therapy (PDT), Liu et al. (2021) [72] formed a neutral Ir (III) complex with a distyryl boron dipyrromethene (BODIPY-Ir), which was found to be effective in instigating the production of reactive oxygen species and onset of hyperthermia to potentiate in vivo tumor suppression. As depicted in Figure 23, through the micellization of BODIPY-IR to form “Micelle-Ir”, BODIPY-Ir (compound **89**), forms J-aggregates within Micelle-Ir, to boost both singlet oxygen generation and photothermal effect to induce severe cell apoptosis; this results in a disruption of the cell cycle and spontaneously generates programmed cell suicide, in which cell degradation is aided by lysosomes [70,71]. When dye molecules aggregate in a parallel fashion with plane-to-plane stacking to form a sandwich-type arrangement, this is known as a H aggregate and causes a blue-shifted absorption—a hallmark example of dye quenching at high concentrations. Conversely, when dye molecules orient in a head-to-toe arrangement (end-to-end stacking), a lower transition energy is exhibited compared to the free dye, resulting in a red-shifted absorption.

**Scheme 12:**
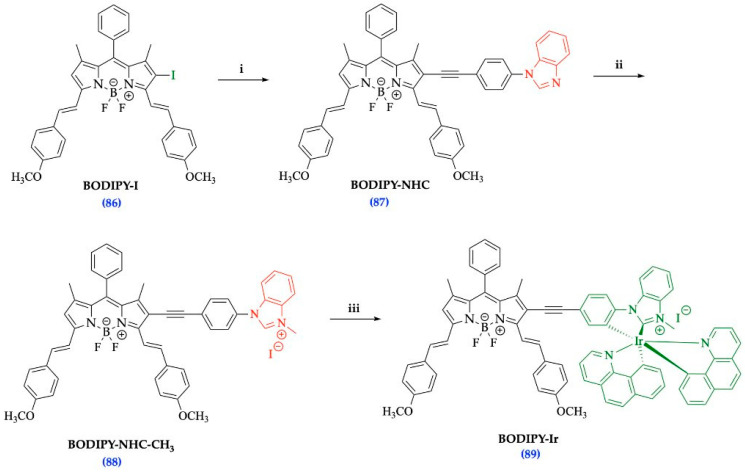
Synthetic route for BODIPY-Ir, a BODIPY species used in forming J-type aggregates with self-assembling micelles to induce cellular apoptosis of tumor cells via reactive oxygen species (ROS) generation [72]. Compound **86** was mono-iodinated to create a synthetic handle for C-C coupling to install an acetylene group, which was further clicked to yield triazole derivative **87**. Compound **87** was subjected to alkylation to create a quaternary nitrogen, which was then subjected to complexation with [Ir(benzo[*h*]quinoline)2(*μ*-Cl)]_2_ to yield compound **89**. This figure was reproduced with permission from Liu et al., Highly Efficient Far-Red/NIR-Absorbing Neutral Ir (III) Complex Micelles for Potent Photodynamic/Photothermal Therapy; published by [Wiley-VCH GmbH], (2021) [72].

As shown in Scheme 12, compound **86** was subjected to Suzuki coupling with a benzo[*d*] imidazole moiety to obtain compound **87**. Then, compound **87** was subjected to methylation in the presence of tetrahydrofuran and excess iodomethane to obtain compound **88**. Finally, compound **88** was heated to reflux under an inert nitrogen atmosphere and treated with Ag_2_O and [Ir(benzo[*h*]quinoline)2(*μ*-Cl)]_2_ to obtain the cyclometalated ligand-enabled Aza-BODIPY fluorophore **89** [72]. As depicted in Figure 23, fluorophore **89** was then subjected to ultrasonication with poly (ethylene glycol)114-poly (*ɛ*-caprolactone)_60_ (PEG-PCL) to form Micelle-Ir. Upon nanoencapsulation, it was found that Micelle-Ir exhibited both further amplified light-to-ROS and heat conversion, promoting both photothermal and photodynamic processes. Due to the addition of the Ir (III) complex, the Micelle-Ir displayed a 0.15 quantum yield and a 29% photothermal conversion efficiency (PCE), promoted by the clathrin-mediated energy-dependent endocytosis with the lysosome that promoted negligible dark cytotoxicity [72].

### 6.7. Advances in Cell Tracking and Cancer Detection Applications

**Figure 24:**
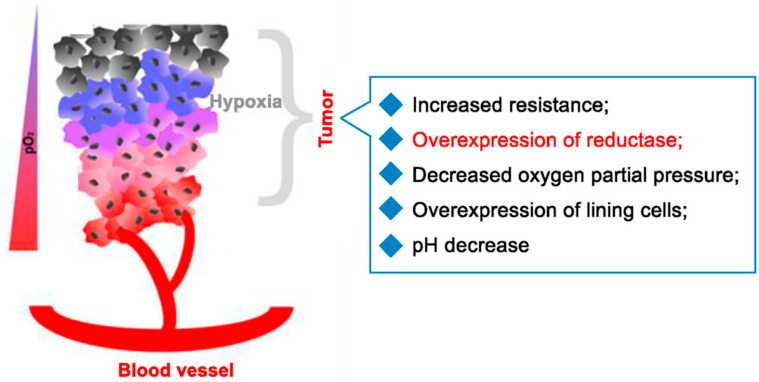
Characteristics of tumor hypoxia, in which hypoxia typically accelerates bio-reductive reactions, causing the increased intracellular expression of reductases, which function as critical biomarkers for assessing the degree of tumor hypoxia. This figure was reproduced with permission from Gai et al., BODIPY-based probes for hypoxic environments; published by [Elsevier], (2023) [77].

**Figure 25:**
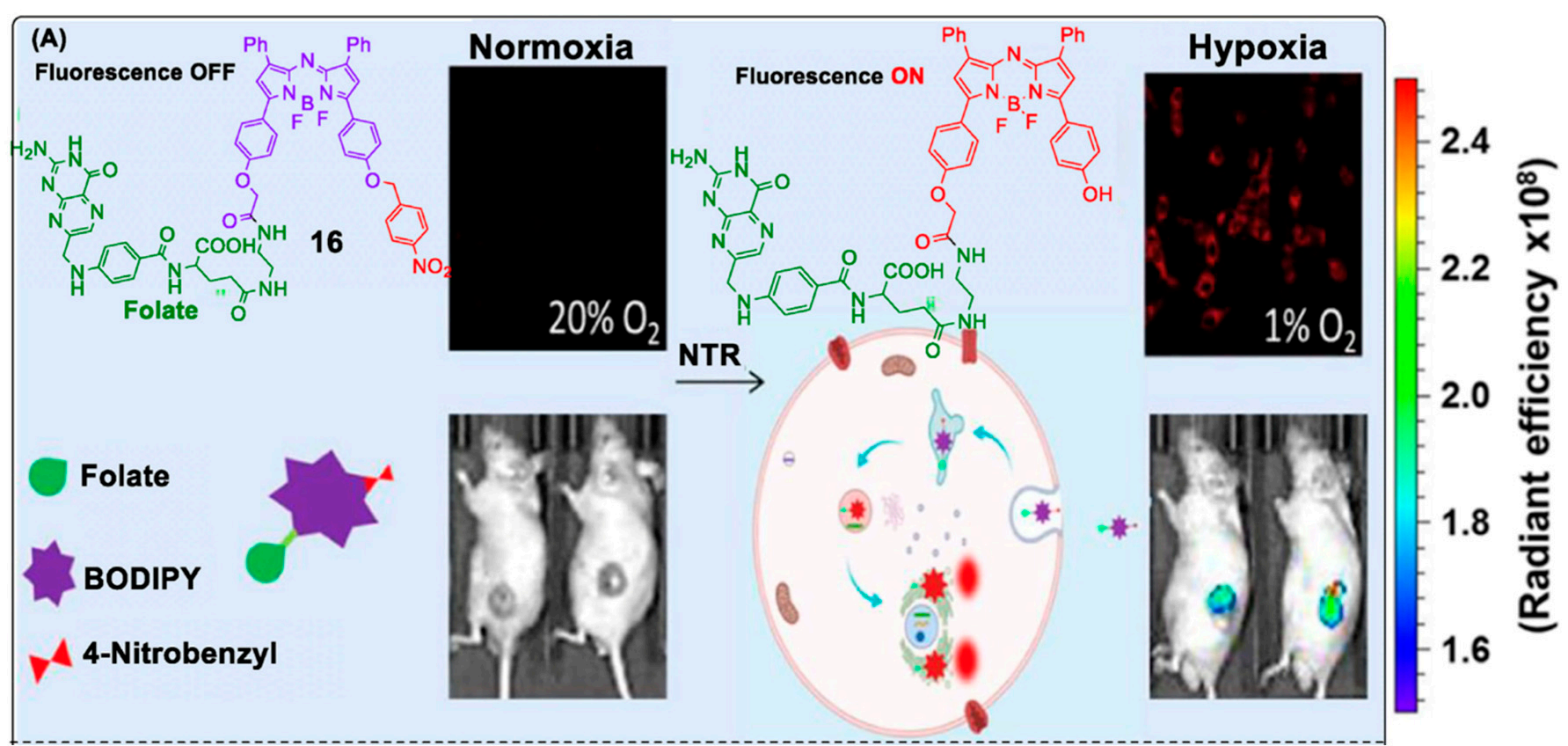
Example of BODIPY conjugation to folate as a means of creating a turn-on fluorophore. This figure was reproduced with permission from Gai et al., BODIPY-based probes for hypoxic environments; published by [Elsevier], (2023) [77].

Advances in the development of photoactivable fluorophores have encompassed tracking cells through the visualization of labeled organelles with excellent localization precision [73,74], especially in tumor detection. However, most synthetic dyes developed for subdiffraction visualization exist as cyanine and xanthene derivatives [75]. Thus, Zhang et al. (2020) designed far-red photoactivable BODIPYs for the super-resolution imaging of live cells for photoactivated localization microscopy (PALM) [76]. In addition, asymmetric anthracene-fused BODIPY dyes synthesized by Yang et al. (2015) conferred a small energy gap (1.81 eV), a large Stokes shift, and high photostability, with extensive π conjugation attributed to red-shifted emission with a quantum yield of 40% and an enlarged Stokes shift of 1425 cm^−1^ (53 nm) in CHCl_3_ [24]. Moreover, cancer tumors are inherently hypoxic environments; thus, BODIPY-based probes leverage their relative insensitivity to pH to be applied to sense hypoxia in vivo and in vitro through fluorescence transformations and recognition mechanisms following interaction with targeted bio-reductive markers in hypoxic regions, as shown in Figure 24 and Figure 25 [77]. In Figure 25, the installation of folate as a targeting moiety serves to advance the criteria outlined in Figure 24. In Normoxia (or normal O_2_ levels), molecular oxygen can serve to quench triplet excited states of fluorophores, which decreases fluorescence. However, when less oxygen is present (hypoxia), the normoxia quenching pathway is suppressed, resulting in a greater proportion of the fluorophore excited state undergoing radiative relaxation, causing fluorescence to increase. With the specific conjugation to folate, folate receptors are overexpressed, and the folate-tagged fluorophore selectively internalizes in hypoxic tumor cells, allowing for accumulation of the dye where oxygen levels are low, improving bioimaging capabilities [77].

**Figure 26:**
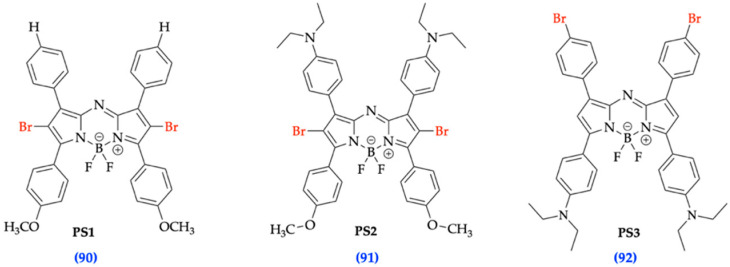
Structures of PS1, PS2, and PS3 are synthesized as a rationale-based design to improve ^1^O_2_ yields of photosensitizers through the introduction of bromine or iodine into the dye core [74].

In 2002, O’Shea and colleagues investigated where heavy-atom substituted fluorophores—compounds **90** (PS1), **91** (PS2), and **92** (PS3), as shown in Figure 26—were synthesized to leverage heavy atom substitutions at the pyrrole ring rather than at the characteristic peripheral tetra (4) phenyl peripheral aryl rings as a means of improving fluorescence quantum yields. PS1 proved to be an exciting PS through Phase 1 clinical trials in tumor models; it showed that direct bromo substitution on the aza-BODIPY core resulted in efficient ^1^O_2_ generation, whereas PS2, bearing a pH-responsive amine receptor, proved to confer deleterious unwanted side effects as a non-selective PS due to accumulation in BOTH tumor and adjacent, healthy tissues [74].

**Scheme 13:**
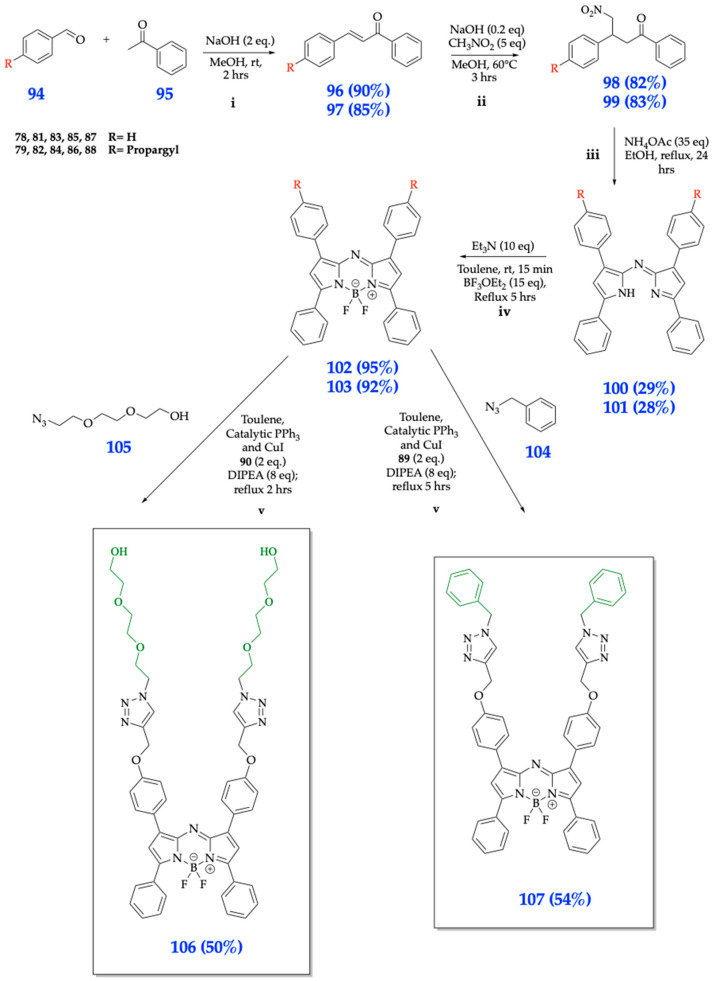
Synthetic scheme for stepwise synthesis of triazole conjugated Aza-BODIPYs, resulting in fluorophores **106** and **107** [74].

Hlogyik et al. (2023) [74] synthesized triazole-based Aza-BODIPY derivatives that were obtained via Cu(I)-catalyzed azide-alkyne cycloaddition (CuAAC) as the key step towards functionalization for in vitro photodynamic activity as photosensitizers. By leveraging the innate excellent photophysical properties conferred by the Aza-BODIPY scaffold, they sought to exploit “click chemistry” to install (*N*-benzyltriazolyl) or azido (triethylene glycol) side chains to improve biological activity; thus, two novel derivatives (**91** and **92**) were synthesized and proved to be optically beneficial demonstrations of the well-established methodology of using copper (I) iodide (CuI) as a catalyst and triphenylphosphine (PPh_3_) as an accelerating ligand, as shown in Scheme 13 [74].

Limitations of BODIPY dyes include a limited synthetic availability and scope, low fluorescence quantum yield, poor or little to no emission maxima, and optical instability in certain solvents, particularly aqueous media [74]. Through evaluation of photophysical characterization, in vitro dark and light cytotoxicity studies, and evaluation of in vitro ROS generation of **106** and **107**, their candidacy for being photosensitizers was evaluated [74]. However, Table 6 displays the photophysical properties of **106** and **107** in DMF, where all values are the same except for **107**, which possesses a higher molar extinction coefficient.

**Figure 27:**
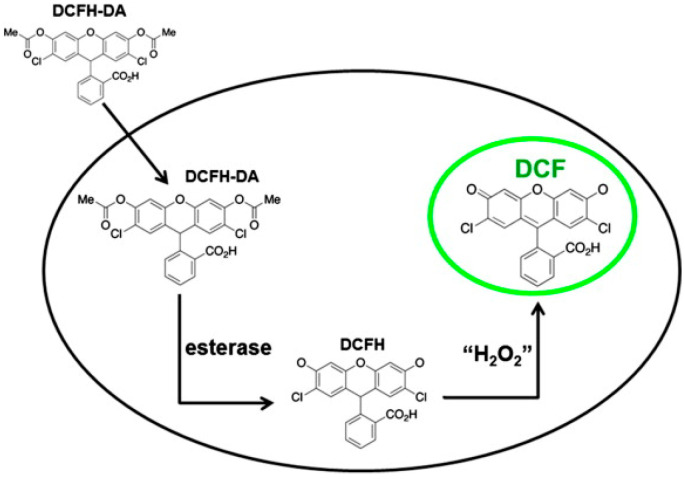
Mechanism of DCF-DA responding to oxidative stress analytes to form fluorescent 2′,7′-Dichlorofluorescin (DCF). Reproduced with permission from Aranda et al., Dichloro-dihydro-fluorescein diacetate (DCFH-DA) assay: a quantitative method for oxidative stress assessment of nanoparticle-treated cells; published by [Elsevier], (2013) [78].

Subsequent evaluation of in vitro dark- and light-induced toxicity of aza-BODIPY dye was conducted by subjecting fluorophores **102, 103, 106**, and **107**, as shown in Scheme 13, to the epidermoid carcinoma cell, A431. The two triazolyl aza-BODIPY derivatives, namely tetraphenyl compound **102** (IC_50_: 0.96 μM) and its *bis*-triazolyl-TEG counterpart **106** (IC_50_: 3.66 nM), resulted in efficient cell killing when irradiated, with the latter being 268-fold more active [74]. The introduction of the triazole moiety was added to increase anticancer potential and coupled with investigation of in vitro ROS generation, shown in Figure 27, the potential mechanism for light-induced toxicity is posited by the passive accumulation of molecular ROS indicator, 2′,7′-Dichlorofluorescin diacetate (DCFH-DA) within the cells.

**Figure 28:**
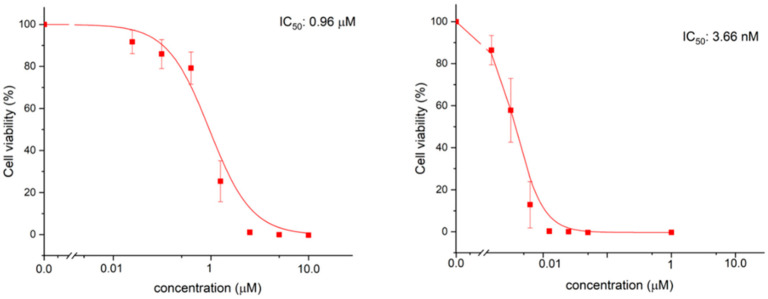
Left to Right: Concentration-dependent light-induced toxicity of compounds **102** and **106** with calculated IC_50_ values shown. Reproduced with permission from Hlogyik et al., Synthesis and in vitro photodynamic activity of aza-BODIPY-based photosensitizers.; published by [RSC Publishing], (2023) [74].

Upon illumination, DCF-DA is deacylated by cell esterases to form dichlorofluorescein (DCFH) and ROS. Hydrogen peroxide results in the formation of fluorescent product 2′,7′-Dichlorofluorescin (DCF), as shown in Figure 27 [74,78]. In conjunction with the reported phototoxicity levels of **102** and **106**, a concomitant increase in ROS levels was reported. However, the cells incubated with non-toxic **103** and **107** displayed a small increase in ROS levels compared to the cells illuminated in the absence of the dyes. Similarly, Figure 28 displays the advantage of improving the concentration-dependent light-induced toxicity through click chemistry to install triazole groups. Compared to parent fluorophore **102**, which possessed an IC_50_ value of 0.96 µM, the progeny fluorophore after CuACC resulted in a nearly 262x increase in IC_50_, with fluorophore **106** having an IC_50_ value of 3.66 nM. As a result, compound **106**, operating in the nanomolar range, serves as a promising candidate for the design of a new, selective, and heavy-atom-free scaffold for photosensitizers, as shown in Figure 28.

### 6.8. Boron Functionalization: Forming Linker-Free NIR Aza-BODIPY Glutamine Conjugates

**Scheme 14:**
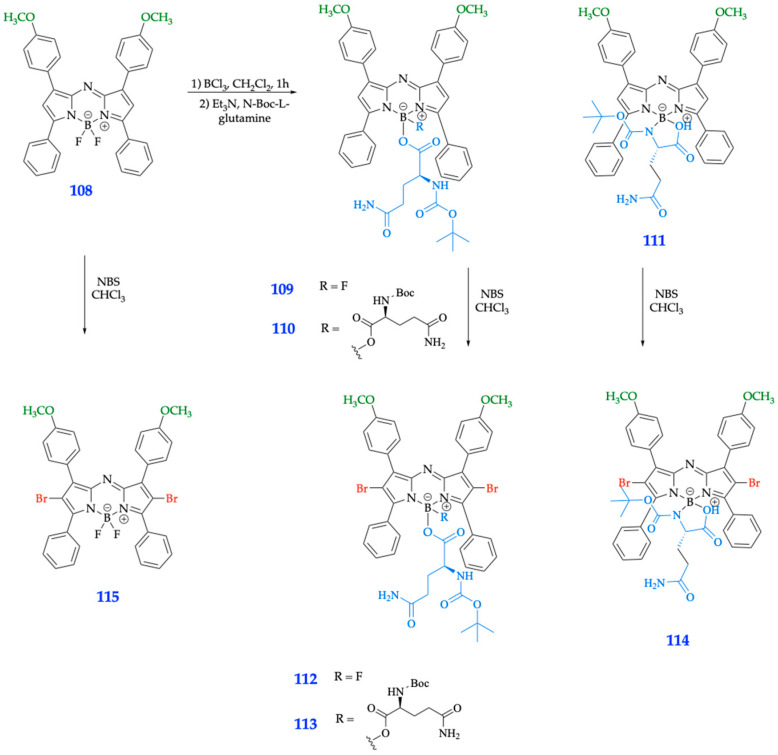
Synthesis of Aza-BODIPY-Gln conjugates **109–114.** This scheme was redrawn but reproduced with permission from Wang et al., Linker-Free Near-IR Aza-BODIPY-Glutamine Conjugates Through Boron Functionalization.; published by [Wiley], (2020) [79].

Impinging on the advents of the introduction of heavy atoms at the 2 and 6 positions on the Aza-BODIPY core as a means of facilitating intersystem crossing and improving triplet state, Wang et al. (2020) deviated from the strategy of modifying the tetraphenyl core and instead developed a one-pot reaction methodology for introducing amino acids onto the boron atom to produce linker-free BODIPY-glycine conjugates, as shown in Scheme 14 [79]. L-glutamine is the most abundant naturally occurring amino acid in humans and plays an integral role in vivo as a nitrogen shuttle for circulating ammonia as well as for protein and glutathione synthesis, energy production, and is beneficial for minimizing infection in trauma and surgery patients [79].

Upon investigating optical spectroscopic and theoretical computational properties, compounds **108** and **109** exhibited increased cytotoxicity upon exposure to a low light dose of (≈1.5 J/cm^2^), whereas the rest of the conjugate fluorophores did not show increased cytotoxicity in response to irradiation. Computational studies elucidated that the 2,6-dibrominated aza-BODIPYs **112, 114**, and **115** showed significantly higher comparative singlet oxygen quantum yields than the corresponding 2,6-unsubstituted aza-BODIPYs **108**, **109**, and **111**, determined using 1,3-Diphenylisobenzofuran (DPBF) as an acceptor, as shown in Scheme 14 [79].

As shown in Table 7, the results are also indicative that the cytotoxicity and the ability of aza-BODIPYs to produce singlet oxygen are not solely contingent upon the presence of bromine atoms at the 2,6-positions but also vary based on the number and substitution of the glutamine units attached to the boron atom. Notably, it is seen that the formation of a cyclic N, O-bidentate aza-BODIPY-Gln, as in compounds **111** and **114**, induces the highest cytotoxicity, while substitution of the boron atom with two glutamine groups and the presence of 2,6-bromines leads to a significant decrease in singlet oxygen production [79]. As a result, Aza-BODIPYs with cyclic N, O- O-bidentate glutamine on boron displayed the highest cytotoxicity among all aza-BODIPY conjugates, making them promising bifunctional anticancer agents, while substitution of the boron atom with two glutamine groups and the presence of 2,6-bromines leads to a significant decrease in singlet oxygen production.

## 7. Biosensing, Chemosensing, and pH Sensing Applications

**Figure 29:**
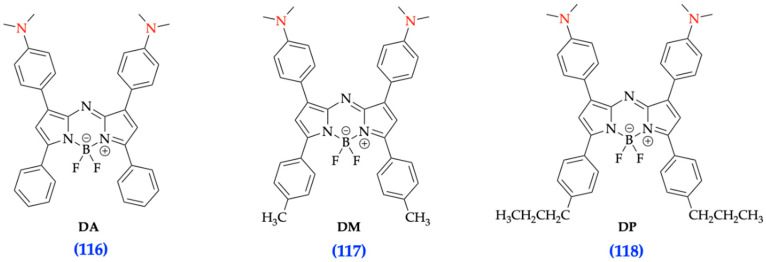
Aza-BODIPYs **116** DA (R = H), **117** DM (R = CH_3_), and **118** DP (R = CH_2_CH_2_CH_3_) were developed for metal sensing via dimethylaniline (NMe_2_) substituents [82].

**Figure 30:**
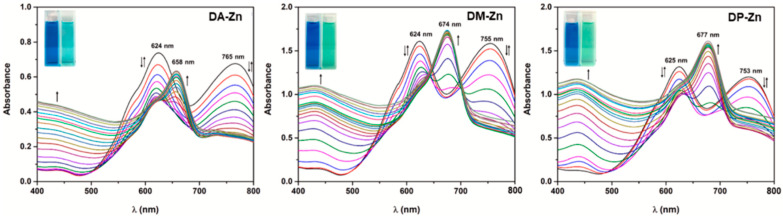
UV–vis spectra of compounds **116** (DA), **117** (DM), and **118** (DP) (0.026 mM) titrated with Zn^2+^ at various equimolar ratios. The inset shows the color changes in the initial aza-BODIPY solution (blue) and the final color of the aza-BODIPY-Zn mixture (light emerald-green) [82]. This figure was reproduced with permission from Luangphai et al., Aza-BODIPY-based logic gate chemosensors and their applications.; published by [Elsevier], (2024) [82].

Apart from activation due to environmental effects, enzymes, and nucleic acids, different prominent widespread approaches are used to produce a PS. Photosensitizer activation mechanisms include electrostatic assembly and bond formation, which cleave; self-quenching; and numerous checkpoint-controlled activations that have been mentioned before. Control points in different locations are another approach to photosensitizer activation, successfully functioning as a photosensitizer activation logic controller [80,81]. Luangphai et al. (2024) [82] developed three aza-BODIPYs **116** (DA-Zn), **117**(DM-Zn), and **118** (DP-Zn), as shown in Figure 29, bearing dimethylaniline substituents that had Zn^2+^ recognition ability and, upon binding to Zn^2+^, exhibited the propensity for the fluorophore to be leveraged as a custom-built colorimeter for the detection of various environmental pollutants. For all three compounds, before the addition of Zn^2+^, two strong absorption bands are observed at ~624 nm and ~760 nm, corresponding to the blue color appearance of them. These bands were attributed to the S_0_–S_1_ transition and therefore exhibited high intensity, while the significantly weaker band located at ~430 nm is attributed to the S_0_–S_2_ transition, as shown in Figure 30 [81,82]. The multiple bands implied that the presence of multiple closely lying electronic states led to these strong charge transfer (CT) transitions. CT involves the movement of an electron from a donor region (electron-rich area) to an acceptor region (electron-deficient area) [81,82].

### Metal Sensing, pH Sensing, and Detection of Reactive Oxygen Species (ROS)

**Figure 31:**
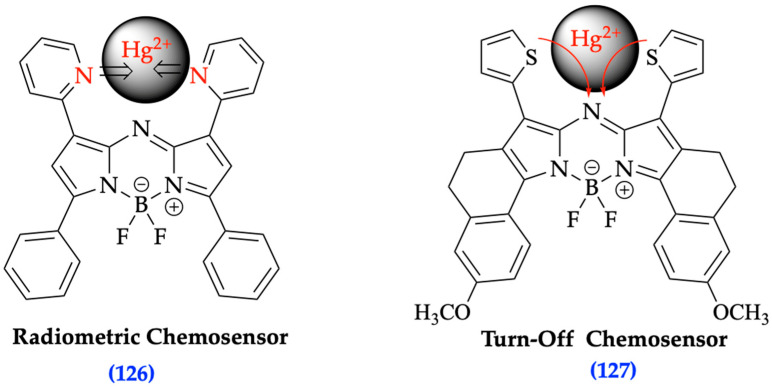
The electron-rich thienyl groups attached to the BODIPY core in compound **111** cause fluorescent quenching because the binding to Hg^2+^ results in an intramolecular charge transfer (ICT) process. This graphic was adapted with permission from Jiang et al., Synthesis of NIR fluorescent thienyl-containing aza-BODIPY and its application for detection of Hg^2+^: Electron transfer by bonding with Hg^2+^; published by [Elsevier], (2015) [83].

For instance, thienyl-containing aza-BODIPY dyes have been successfully synthesized using sensors for heavy metal detection of mercury, as shown in Figure 31, by utilizing the innate electronic configuration and lack of ligand field stabilization conferred by Hg^2+^ to leverage it as a soft acid to accommodate a host of coordination numbers and geometries [83]. Mercury pollution is prominent in contaminated water, and its toxicity stems from the high affinity of thiol groups in proteins and enzymes. Industrial sources of mercury are attributed to coal mining, gold mining, solid waste incineration, fossil fuel consumption, and chemical manufacturing, resulting in the release of inorganic mercury Hg (0) and Hg (II) into the environment. Mercury poisoning has been connected to severe sensory, motor, and cognitive disorders [83].

**Scheme 15:**
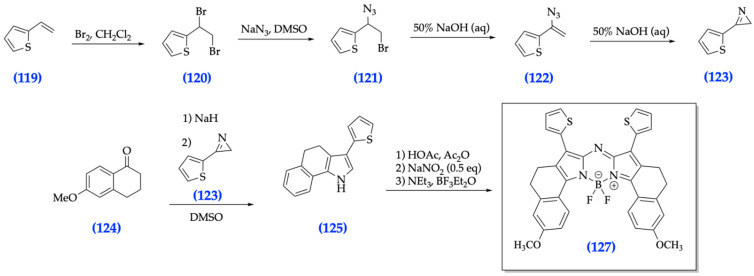
Synthetic development of NIR fluorescent aza-BODIPY **127**, which was found to be a turn-off fluorescent chemosensor. This scheme was redrawn but reproduced with permission from Jiang et al., Synthesis of NIR fluorescent thienyl-containing aza-BODIPY and its application for detection of Hg^2+^: Electron transfer by bonding with Hg^2+^; published by [Elsevier], (2015) [83].

Thus, the detection of mercury ions was of interest using aza-BODIPY dyes. The novel synthesis of this aza-BODIPY dye was obtained by utilizing the starting material of 2-vinylthiophene to obtain 3-(thiophene-2-yl)-2H-azirine required in the synthesis of a pyrrole. As displayed in Scheme 15, once the pyrrole was obtained, it was treated under HOAc, Ac_2_O, and NaNO_2_, followed by the traditional BODIPY synthesis using the complex of BF_3_•EtO_2_-Et_3_N to yield fluorophore **127** [83]. The thienyl-containing aza-BODIPY dye demonstrated optical properties of absorbance (λ_abs_ = 760 nm) and emission (λ_em_ = 782 nm) in the NIR, and with high extinction coefficients of (136,000 M^−1^ cm^−1^) in fluorescence. However, under physiological conditions of 10 µM of HEPE buffer at pH 7.3, the aza-BODIPY dye was found to be turned off with the binding of mercury ions. This observation is due to electron-withdrawing groups, which are part of the fluorophore π-system and involved in cation binding; the charge separation in the excited state is more stabilized than in the ground state, which results in the reduction in the S_0_-S_1_ gap. As a result, fluorescence quenching of the electrons from the electron-rich thienyl group to the BODIPY core is transferred after binding with the mercury ions, known as an intramolecular charge transfer (ICT). Thus, this water-soluble aza-BODIPY dye was tested in the presence of other metal ions such as Na^+^, K^+^, Mg^2+^, Ag^2+^, Co^2+^, and others to see the effects on fluorescence intensity, which resulted in no significant difference in energy. Compared with compound **126**, which binds to mercury and exhibits two analyte absorption bands upon binding to the heavy metal, compound **127** is a turn-off chemosensor that results in a reduced fluorescence signal upon binding, as shown in Figure 31.

**Figure 32:**
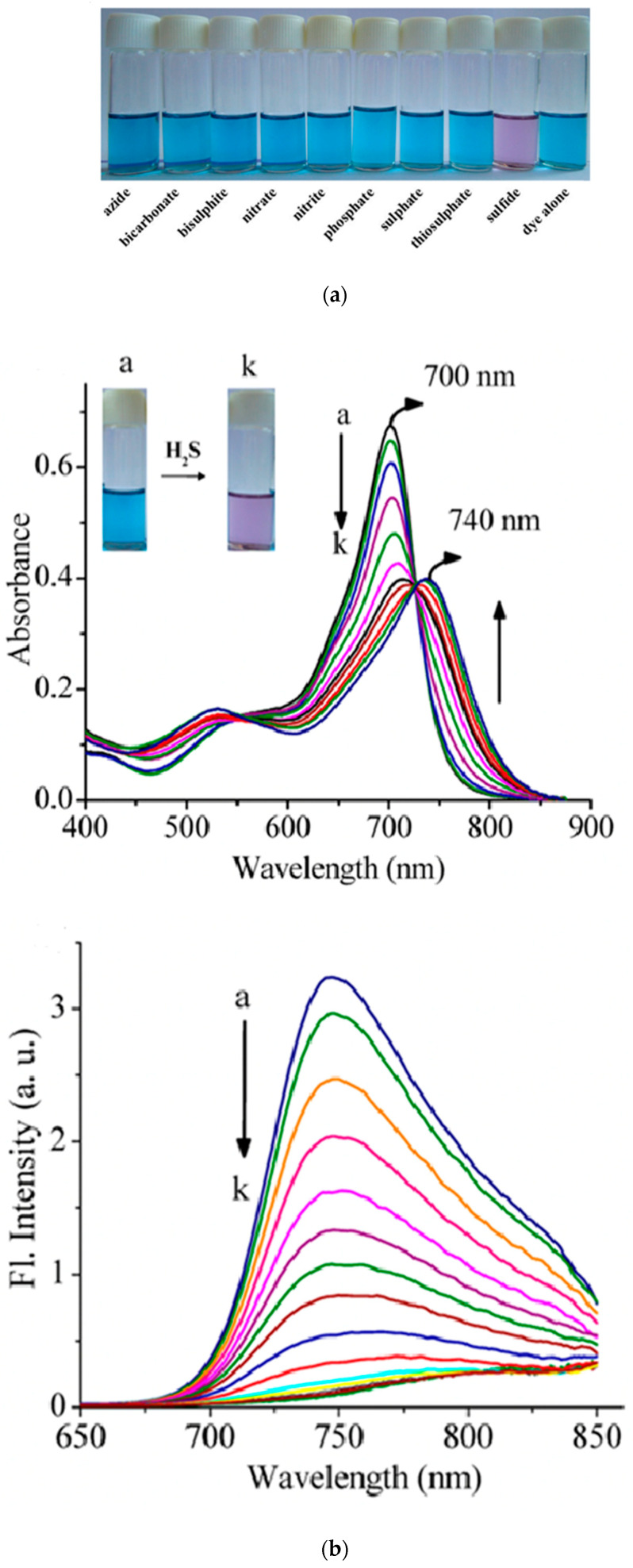
Left: (**a**) Colorimetric response of the azido-containing probe towards various analytes; Right: (**b**) changes in absorption and emission spectrum of azido-containing probe. These figures were reproduced with permission from Adarsh et al., Sensitive Naked Eye Detection of Hydrogen Sulfide and Nitric Oxide by Aza-BODIPY Dyes in Aqueous Medium; published by [American Chemical Society] (2014) [85].

Among the myriad biomedical applications of Aza-BODIPY dyes, they are widely employed as biosensors for the detection of hydrogen sulfide (H_2_S), nitric oxide (NO), and even in pH sensing, specifically in mitochondria targeting [84]. Adarsh et al. (2014) [85] successfully reacted to hydrogen sulfide with three new aza-BODIPY derivatives containing azido (N_3_), amino (NH_2_), and dimethylamino (NMe_2_) groups to detect hydrogen sulfide via color change. Researchers elucidated the detection of H_2_S via color change when the aza-BODIPY probe containing the azido (N_3_) group changed color from bright blue to purple at a sensitivity threshold of 0.5 ppm of H_2_S, as shown in Figure 32a. This color change was discovered to have been caused by the reduction in the azido group substituent on the Aza-BODIPY probe to an amino group via nitric oxide. Similarly, the colorimetric change is marked by the appearance of an isosbestic point at 740 nm in the absorption spectrum and the decrease in fluorescence emission intensity, respectively, as displayed in Figure 32b [85]. In addition, Merkushev et al. (2022) discovered that the primary reason for the solvatochromic effect surpassing the expected value of 5–8 nm for classic BODIPY dyes, and presenting as 15–20 nm, was due to solvent-induced stabilization due to the van der Waals interactions, the formation of hydrogen bonds, and/or the stabilization of the excited states of a solute by the solvent [86].

**Figure 33:**
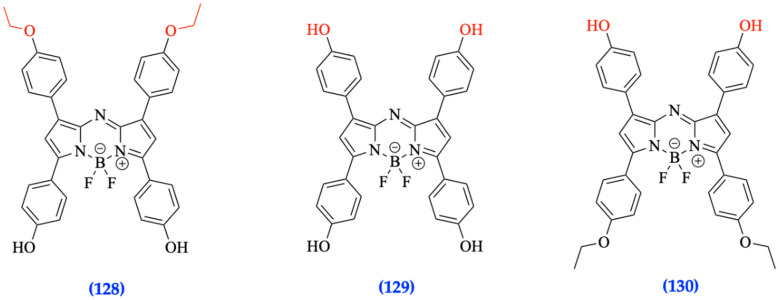
Hydroxylated Aza-BODIPY fluorophores **128, 129**, and **130** [87].

**Figure 34:**
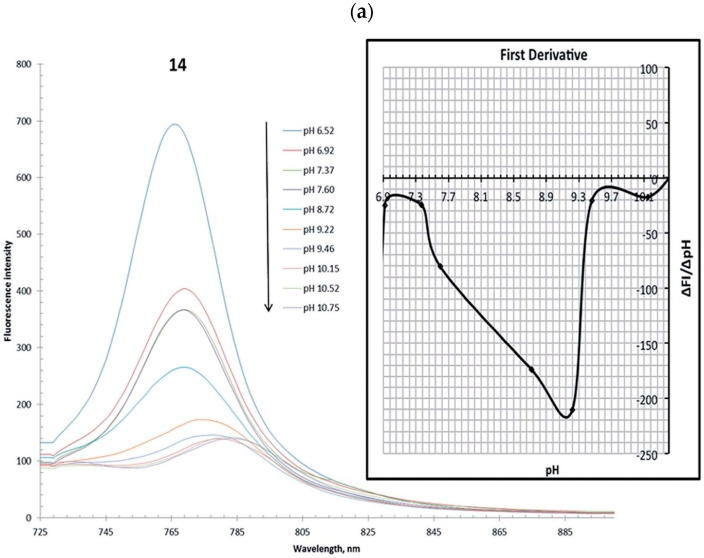

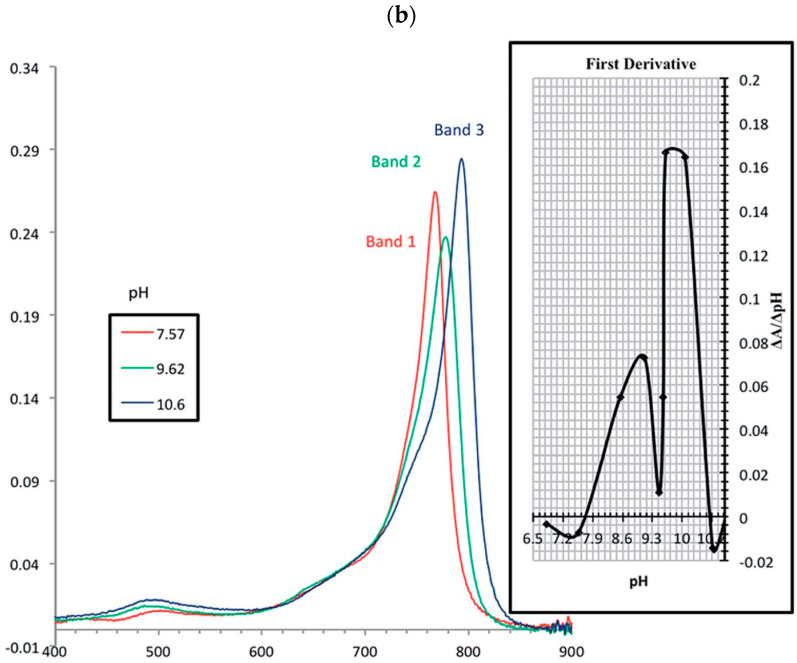
Left: (**a**) pKa analysis of phenolic fluorophore **128** as exhibited by decreasing fluorescence intensity as pH increases and subsequent computation of pKa through the 1st derivative method to determine the inflection point to yield a determined pKa of 10.1 [87]. Right: (**b**) Repeated pH titration of fluorophore **128** computed to yield the 1st derivative of the change in absorbance with respect to the change in pH to yield a pKa value of 10.6. These figures were reproduced with permission from Henary et al., Hydroxylated near-infrared BODIPY fluorophores as intracellular pH sensors; published by [RSC Publishing] (2014) [87].

With regard to pH-sensing capabilities, Henary et al. (2014) devised hydroxylated NIR-BODIPY fluorophores and discovered that the pKa of the aza-BODIPY derivatives can be tuned from 9.10 to 10.85 by changing the position of the hydroxyl groups, as displayed in Figure 33, and exhibited a bathochromic shift in the absorbance maximum wavelength upon deprotonation of the hydroxyl substituent [87]. However, fluorophore 128 was discovered to be a lead compound for further optimization of developing pH-sensitive fluorophores. Through pKa analysis of the plotting of the first derivative with respect to changes in the fluorescence intensity and subsequent changes in pH, an inflection point was computed, as shown in Figure 34a. The significance of this inflection point indicates that at a pH of 10.1, there was no observed change in fluorescence intensity upon inducing a change in pH. As a result, it was logically surmised that this phenomenon must be due to the deprotonation of the phenol group, with the pKa value corresponding to 10.1 [87]. To validate this deductive premise, the pH titration was also performed by computing the change in absorbance intensity with respect to changing pH, where a similar pKa value of 10.6 was obtained, as displayed in Figure 34b [87].

**Figure 35:**
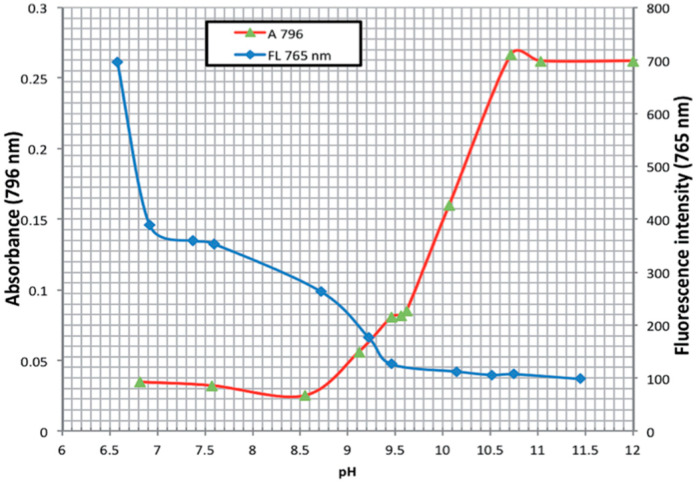
Radiometric pH profile of fluorophore **128**. These figures were reproduced with permission from Henary et al., Hydroxylated near-infrared BODIPY fluorophores as intracellular pH sensors; published by [RSC Publishing] (2014) [87].

Finally, to determine the true pKa of fluorophore **128**, a radiometric profile was computed by overlaying the increase in absorbance at 796 nm and the decrease in fluorescence emission at 765 nm, leading to an intersection at pH 9.20, which was deemed the true pKa of the compound, as shown in Figure 35.

In continuation, pH-sensitive probes with hydroxyl groups in the meta-position of a phenyl substituent can be used for facilitating “on-off” fluorescence responses by eliminating the conjugation to the chromophore [65,88]; Liu et al. (2015) developed an application of the Boronic Acid Functionalized Aza-Bodipy (aza-BDPBA) dye for developing a glucose assay to be responsive to hydrogen peroxide [89]. ROSs are the major source of oxidative stress in cells, alongside RNSs (reactive nitrogen species). Both are highly reactive but RNSs tend to be less damaging to cellular components than ROSs [12,90,91]. Advancements in using BODIPY for tracking mitochondria have entailed conjugating cations to the molecules, with cationic triphenylphosphine (TPP^+^) and pyridinium commonly used because TPP^+^ enhances lipid solubility. When attached with TPP^+^ and ethylene glycol, it enhanced photothermal and photoacoustic effects to target mitochondria more precisely and efficiently. Also, TPP^+^ exhibited high stability and low cytotoxicity [12,90,91].

**Figure 36:**
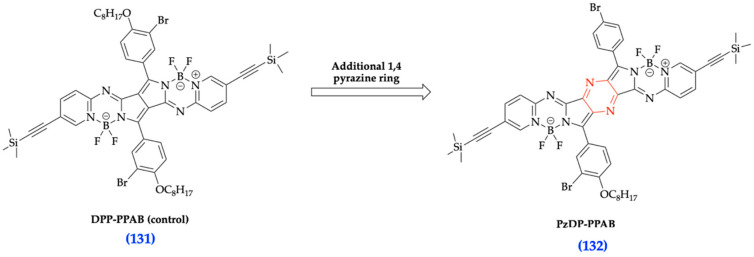
Compounds **131** (control) and compound **132**, which displayed NH_2_NH_2_ and ClO^−^, induced distinct chromophore reactions to break the fluorophore upon binding to N_2_H_4_ and ClO^−^ [92].

**Figure 37:**
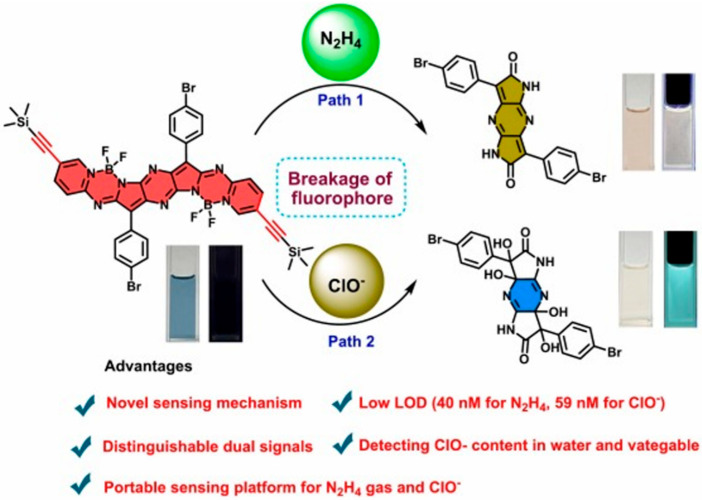
Depictions of colorimetric responses of DPP-PPAB (**131**) and PzDP-PPAB (**132**) and N_2_H_4_ and ClO^−^ [92]. This figure was reproduced with permission Zhang et al. (2025), A portable sensing platform using a novel dipyrrolopyrazinedione-based aza-BODIPY dimer for highly efficient detection of hypochlorite and hydrazine; published by [Elsevier], (2015) [92].

Notably, Zhang et al. (2025) [92] devised a portable sensing platform using a novel dipyrrolopyrazinedione-based Aza-BODIPY dimer (PzDP-PPAB, compound **132**) to be highly efficient at detecting hypochlorite (ClO^−^) and hydrazine (N_2_H_4_). Due to the limitations in current fluorescence detection of these two analytes, current methods exhibit slower and minor spectral changes, require high detection limits, and confer poor selectivity. As a result, the newly developed pyrrolopyrrole aza-BODIPY (DPP-PPAB) control compound **131** was synthesized from heteroaromatic amines and diketopyrrolopyrrole (DPP) to form a precursor intermediate compound, PzDP, which is not displayed. Then, through an imination reaction of electron-deficient PzDP precursor, activated by TiCl_4_ with heteroaromatic amine for the first time, as shown in Figure 36, an additional 1,4 pyrazine ring is added to yield fluorophore **132**, as shown in Figure 36 [92]. When comparing the spectral response of PzDP-PPAB, compound **132**, and DPP-PPAB, compound **131**, to N_2_H_4_ and ClO^−,^ as shown in Figure 35, PzDP-PPAB **132** exhibited the potential to be utilized as an efficient fluorescent probe for simultaneous detection of N_2_H_4_ and ClO^−^, with distinguishable color change and a remarkable fluorescence “ON” behavior, by introducing an electron-deficient 1,4-pyrazine ring-enhanced chromophore reaction strategy, as shown in Figure 37. In the case of N_2_H_4_, the slow decay of shoulder absorption at 588/628 nm in the NIR and the appearance of a new band at 378 nm were shown with the increase in reaction time. The spectra remained invariant after 30 min [92]. On the contrary, the presence of ClO^−^ induced the shoulder absorption band to decay quickly, and the new band at 351 nm gradually strengthened [92].

Similar efforts to advance environmental detection of Fe^3+^ were conducted by Azizi-Khereshiki et al. (2025), where colorimetric olive oil (OL)-Ag nanoparticles exhibited excellent selectivity for Fe^3+^, displaying a low detection limit (LOD) of 0.81 μM and a limit of quantification (LOQ) of 2.7 μM [93]. Ahmed and Henary (2025) also advanced the inception of metal sensing among dimeric fluorophores through the dimerization of heptamethine monomeric dyes to detect Cu^2+^, where it was reported that a survey of eight dimeric heptamethine fluorophores did not exhibit any spectral changes in response to Ag^+^, Li^+^, Na^+^, K^+^, Cr^3+^, Ni^2+^, Co^2+^, Zn^2+^, Hg^2+^, and Ca^2+^, but did towards Cu^2+^, rendering the fluorophores selective for Cu^2+^ detection [94].

**Figure 38:**
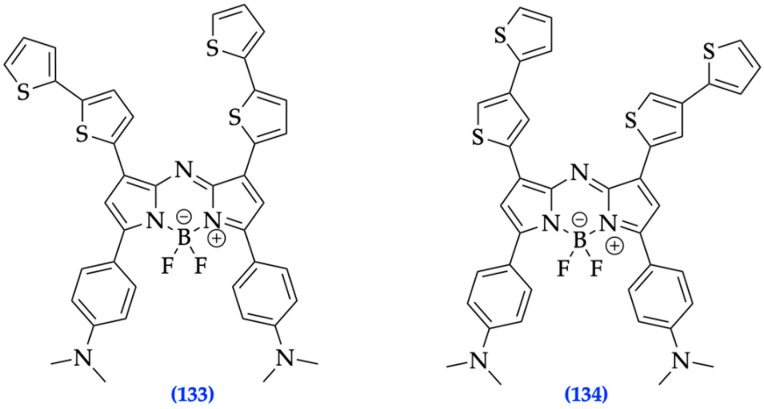
Bithiophene fluorophores **133** and **134** synthesized through O’Shea’s route [95].

**Figure 39:**
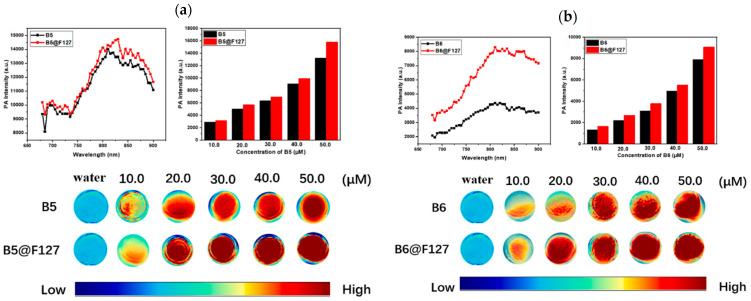
Comparison of photoacoustic signals for fluorophores **133** (B5) shown in (**a**) and **134** (B6) as shown in (**b**) in both water and encapsulated in Pluronic F127 to create nanoparticle micelle structures. This figure was reproduced with permission from Yong et al., Design, synthesis, and characterization of pH-responsive near-infrared bithiophene Aza-BODIPY; published by [Elsevier], (2023) [95].

Alongside the advances in pH sensor development, researchers have sought to leverage new heterocycles to design probes that simultaneously exhibit detection modalities through pH-sensitive responses, while also displaying therapeutic modalities through the tuning of photoacoustic/fluorescence imaging properties and shifting into the NIR-II window. For instance, Yong et al. (2023) [95] developed near-infrared bithiophene Aza-BODIPY probes, compounds **133** and **134**, which differ in the installation of the bithiophene moiety at the 2 and 3 positions, respectively, as shown in Figure 38. Compounds **133** and **134** exhibited the ability to produce singlet oxygen (^1^O_2_) at pH = 5, but this was significantly enhanced at pH = 7, while maintaining excellent photoacoustic properties, as shown in Figure 39a,b, with fluorophores **133** (denoted as B5) and **134** (denoted as B6) alone versus encapsulation with Pluronic F127 to develop nanoparticles [94]. Preliminary pH studies showed fluorophore **133** had better photodynamic performance at pH = 5, whereas the photodynamic performance of **134** was improved under acidic conditions [95].

**Figure 40:**
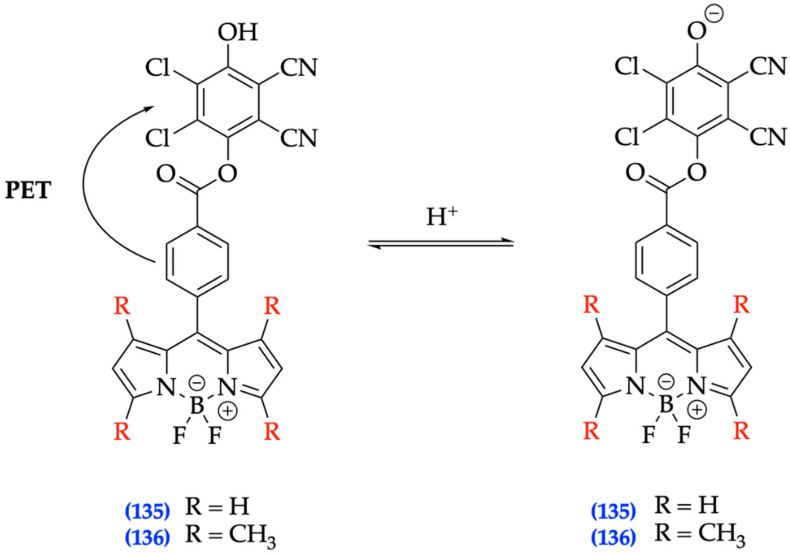
Protonation equilibrium of **135** and **136** resulted in fluorescence in acidic media by PET mechanism [57].

Additional advances made in developing fluorescence pH sensors based on BODIPY structure sensitive in acidic media were conducted by Glavaš et al. (2023) [57], who synthesized two BODIPY phenolic esters, compounds **135** and **136**, as shown in Figure 40, which exhibited pH-responsive fluorescence with quenching of fluorescence by protonation due to photoinduced electron transfer (PET) from the BODIPY core to the phenol substituent. Electrochemical measurements and computational studies validated the feasibility of the PET mechanism in the protonated form of sensor **135** [57].

**Scheme 16:**
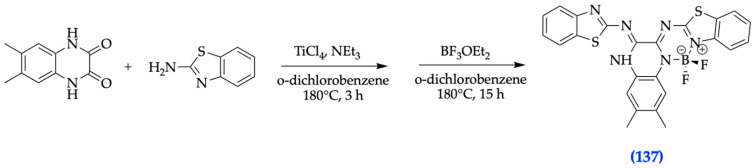
Synthesis of fluorophore **137 [96]**.

**Figure 41:**
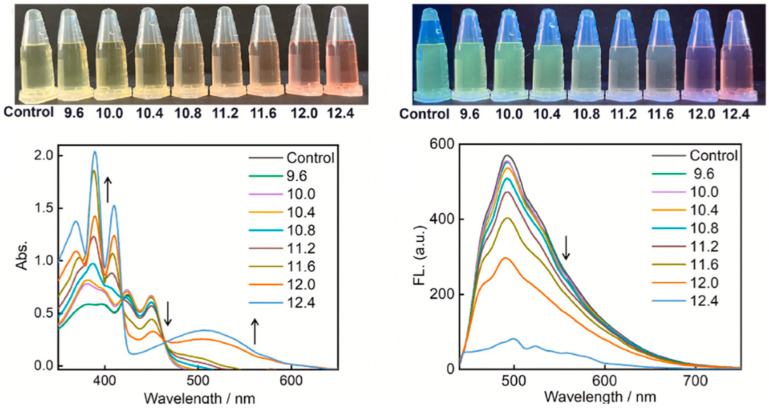
Photographs of probe 137 under daylight top: (**left**) and UV light top: (**right**) at a varying pH from 9.6 to 12.4. Bottom: (**Left**)—Absorption and bottom (**right**)—fluorescence spectra of probe **137** (40 μM) in DMSO/H_2_O solution (9:1, *v/v*) with a varying pH from 9.6 to 12.4 (λ_ex_: 425 nm, Excitation/Emission slit: 10/10 nm). This figure was reproduced with permission from Chen et al., A novel BODIPY-based fluorescent probe for naked-eye detection of the highly alkaline pH.; published by [Elsevier], (2025) [96].

Conversely, in the alkaline pH range, Chen et al. (2025) [96] devised a novel pH-responsive asymmetric aza-BODIPY probe via Schiff base formation, which exhibited a turn-off fluorescence response between pH 9.6 and 12.4, with a computed pKa of 11.65. As shown in Scheme 16, the synthesis begins with a condensation reaction between a dione and aminobenzothiazole unit to reflux in dry dichlorobenzene for 10 min, then TiCl_4_ and TEA are added to form the precursor aza-dipyrromethene intermediate, as evidenced by the imine formation product completed via TLC. Finally, the resulting substrate is subjected to boron complexation [96]. Mechanistically, it was discovered that the probe relies on the deprotonation of the imine group within the aza-BODIPY core to confer an enhanced π-electron conjugation, in which the results of the pH-dependent fluorescence study revealed fluorescence decreasing with increasing pH until pH 12.4, in which the fluorescence was fully quenched [57], as shown in Figure 41. Figure 41 also demonstrates how the pH-response of fluorophore **137** is contingent upon the deprotonation of the imide group, as it significantly affects the absorption spectrum, in regions where the pH increases from 9.6 to 12.4, the intensities of the bands at 425 and 450 nm decrease, while the intensities around 381 nm and 397 nm increase, and a new absorption band emerges at 506 nm, as shown in Figure 41 [96].

## 8. Notable NIR-II Aza-BODIPY Derivatives: Design Strategies, Biomedical Applications, and Photophysical Properties

**Figure 42:**
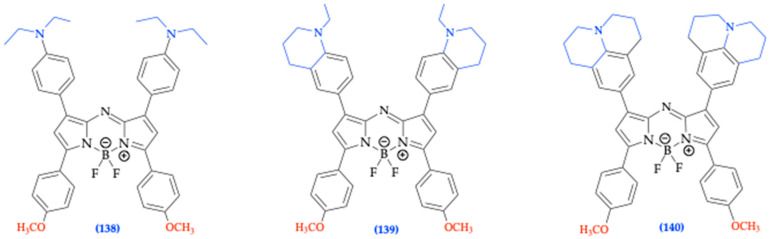
Developed NIR-II Aza-BODIPY fluorophores **138, 139**, and **140**, incorporating 4-(*N, N-*dimethylamino) phenyl, 1-ethyl-1,2,3,4-tetrahydroquinolinyl, and 4-julolidinyl substituents, respectively [97].

**Figure 43:**
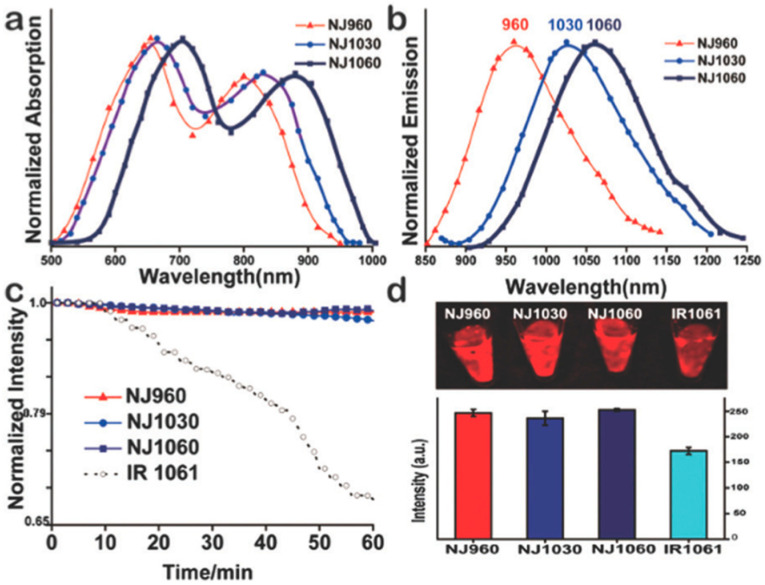
Portrayals of (**a**) normalized absorption spectra, (**b**) normalized emission spectra, (**c**) normalized emission decay, and (**d**) NIR-II fluorescent images of fluorophores **138**–**140** in PBS solution at 808 nm. This figure was reproduced with permission from Bai et al., Novel aza-BODIPY based small molecular NIR-II fluorophores for in vivo imaging; published by [RSC Publishing], (2019) [97].

In 2019, Bai et al. [97] developed a novel aza-BODIPY small molecule NIR-II fluorophore, overcoming a limitation of the traditional facile syntheses. Fluorophores NJ960 (**138**), NJ1030 (**139**), and NJ1060 (**140**) were created by incorporating strong electron-donating groups into the 3 and 5 positions of the aza-BODIPY core, as shown in Figure 42. These modifications were predicated on the premise of using intramolecular charge transfer (ICT) to lower the energy gap of the allowed electronic transitions from the HOMO to LUMO. The photophysical properties of fluorophores NJ960 (**138**), NJ1030 (**139**), and NJ1060 (**140**) displayed in PBS solution (pH = 7.4) containing 20% DMSO as a co-solvent exhibited major absorption bands in the NIR-I region ranging from 651 to 910 nm. The general paradigm exhibited is that the fluorescence spectra of fluorophores NJ960 (**138**), NJ1030 (**139**), and NJ1060 (**140**) erred further into the NIR-II region as solvent polarity increased, with fluorophore **140** bearing julolidine units exhibiting the largest absorbance bathochromic shift, as displayed in Figure 43a–c. Fluorophores **138**–**140** also exhibited superior photostability and brightness after 10 uM concentrations were continuously irradiated with 808 nm, 100 mW cm^−2^ light for 60 min, where their emission intensities were nearly constant and unchanged for the full duration of the 1 h, as shown in Figure 43d [97].

**Figure 44:**
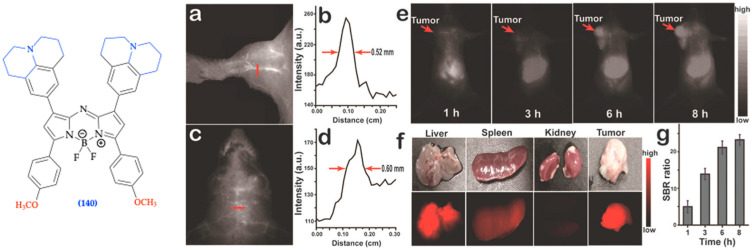
NIR-II images of the mouse hind limb (**a**) and brain (**c**) vasculature of fluorophore **140**. Frames (**b**,**d**) display the emission intensity profile of fluorophore **140**. Frame (**e**) showing the 4T1 breast cancer tumor from hours 1 to 8 after tail vein injection of the fluorophore **140** nanoparticles (NP) under 808 nm laser excitation. Frame (**f**) displays the ex vivo biodistribution of fluorophore **140** in the liver, spleen, kidneys, and tumor at 4 h under an 808 nm laser. Frame (**g**) shows the SBR to This figure was reproduced with permission from Bai et al., Novel aza-BODIPY based small molecular NIR-II fluorophores for in vivo imaging; published by [RSC Publishing], (2015) [97].

Finally, fluorophores **138**–**140** were subjected to in vivo imaging studies, where fluorophore 140 emerged as the lead compound for future optimization citing best cytotoxicity results from the in vitro studies, where fluorophore **140** was encapsulated in nanoparticles and displayed no cytotoxicity in concentrations up to 400 μg/mL [97]; therefore, fluorophore **140** was deemed safe to be used for NIR-II imaging applications in murine models. In vivo imaging biodistribution studies revealed uptake into the hind limb and brain vasculature, where a maximum imaging depth of 8 mm was achieved, as shown in Figure 44a–d, higher than that of any other NIR-I imaging agents, which usually reach about 0.2 mm in depth.

As time reached 3 h, a decreased fluorescence signal was observed in the hind limb vasculature, whereas an enhancement in the abdomen fluorescence signal was observed in the 4T1 tumor within the mice’s left shoulder. Between hours 6 and 8, the fluorescence signal within the abdomen remained constant, whereas the tumor fluorescence signal increased in SBR from 4.9 to 23 (8 h), and reached maximum SBR of 30 at 24 h [97]. Figure 44e shows the ex vivo biodistribution study that corroborates the high fluorescence intensity in the liver, spleen, and tumor, where fluorophore **140** demonstrated preferential accumulation in the liver and spleen [97].

## 9. Conclusions

Thus, the BODIPY class of NIR dyes has gained momentum in advancing the inception of biomedical imaging and cell imaging/tracking. As evidenced by the chemistry and synthetic pathways outlined in this updated review, the synthesis of the BODIPY fluorophores has become increasingly versatile through the years 2014–2025, to the advent of creating novel BODIPY fluorophores and through the C-H arylation of meso-substituted BODIPYs with various bromoarenes. Also, through the three synthetic routes for developing aza-BODIPY fluorophores, derivatives have been fine-tuned in their photophysical properties as well as tumor targeting through conjugation with biomolecules such as biotin and folate. Additionally, BODIPY metal sensing capabilities detect various heavy-metal and anionic analytes, presenting a finite biomedical translational value towards emerging therapeutics. This advent is promising through the modifications to the boron core, by installing new moieties such as cyano and alkyne groups, creating synthetic handles for click chemistry (i.e., addition of triazole and sugar units to aid in cellular surface recognition for binding). Moreover, the addition of cyano groups renders the post-functionalization of the aza-BODIPY and BODIPY fluorophores to be achieved by maximizing photoactive and redox properties prior to functionalizing bioimaging applications. The limitations of venturing deeper into the NIR-II region have begun to be circumvented by C-C coupling and the incorporation of synthetically diverse heterocycles to increase conjugation and planar rigidity. In addition, elucidating information to bridge the gaps of understanding how to achieve desirable optical properties, while avoiding hydrophobicity, and leveraging the advances in rationale-based design for selective analyte detection and pH sensitivity. Future work entails enhancing the in vivo stability by the addition of cationic and anionic groups to facilitate solubility in aqueous regions (blood, plasma, and urine), while maintaining renal clearance, especially through the continued advancement of probing the NIR-II window; further development with nanoparticles is needed to leverage photodynamic, photoacoustic, and photothermal pathways.

## Figures and Tables

**Table 1:** Summary of reported yields and representative compounds for the three synthetic methods of Aza-BODIPY fluorophores [36].

Method	Reported Yield	Representative Compounds	Forms: Asymmetrical or Symmetrical Fluorophores?
O’Shea’s [35]	20–50% [36]	Tetraphenyl Aza-BODIPY Dimethylamino Aza-BODIPY	Both
Carriera’s [37]	60–90% [36]	Cyclized 2,4-diaryl pyrroles complexed with BF_3_OEt_2_	Both
Lukyanet’s [38]	10–30% [36]	Phthalonitrile and aryl Grignard reagents complexed with BF_3_OEt_2_	Symmetrical Only

**Table 2:** Tabulated photophysical properties for compounds **46**, **47**, and **48** in H_2_O/DMSO mixtures (*v*/*v* 999/1) at pH 4.7 (10 mM acetic acid/acetate buffer): [a] fluorescence quantum yield ±15%; [b] fluorescence enhancement factor (FEF = I/I_0_], (±0.2); [c] dissociation constants K_d_; [d] K_d_ value for these cations’ complexes could not be calculated due to low-cation-induced intensity changes [46].

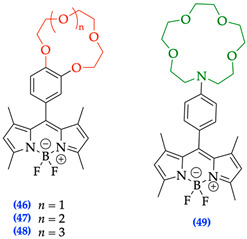					
Compound	λ_abs_ [nm]	λ_em_ [nm]	Φf ^[a]^	FEF ^[b]^	K_d_ ^[c]^ [mM]
**46**	498	506	0.072	-------	-------
**46** + 100 mM Na^+^	498	507	0.073	1.0	---- _[d]_
**46** + 100 mM K^+^	498	507	0.072	1.0	---- _[d]_
**46** + 100 mM Li^+^	498	507	0.072	1.0	---- _[d]_
**47**	498	507	0.008	-------	-------
**47** + 1000 mM Na^+^	498	507	0.056	7.3	276
**47** + 1000 mM K^+^	498	507	0.023	2.8	342
**48**	498	507	0.008	-------	-------
**48** + 250 mM Na^+^	498	507	0.013	1.8	65
**48** + 250 mM K^+^	498	508	0.035	4.7	18

**Table 3:** Photophysical properties of compounds **67** and **68** in CHCl_3_. ^[a]^ Oxazine standard using ethanol as a solvent [45].

Compound	λ_abs_ [nm]	log [ε]	λ_em_ [nm]	Φf %^[a]^
**67**	630	4.80	690	55.4
**68**	625	4.80	678	51.0

**Table 4:** Comparison of reaction times and yields across varying aryl rings [58]. ^a^ Denotes reaction conducted under traditional conventional synthesis; ^b^ denotes reaction conducted in microwave-assisted synthesis.

Entry	Compound	Ar	Reaction Time	Yield (%) of 69	Yield (%) of 70
**1**	A	Phenyl ^a^	24 h	44	17
**2**	B	4-Anisyl	43 h	42	10
**3**	C	4-(Dimethylamino)-phenyl	48 h	---	---
**4**	D	3-Thienyl ^a^	27 h	55	10
**5**	E	Mesityl	43 h	35	0
**6**	F	1-Naphthyl	24 h	20	16
**7**	G	4-Cyanophenyl	48 h	---	---
**8**	H	3-Nitrophenyl	48 h	---	---
**9**	I	Phenyl ^a^	28 h	31	32
**10**	J	Phenyl	46 h	28	18
**11**	K	Phenyl	4 days	13	43
**12**	L	Phenyl	4 days	---	39
**13**	M	Phenyl ^b^	3 h	39	40
**14**	N	3-Thienyl ^b^	3 h	26	---
**15**	O	Phenyl ^b^	3.5 h	28	46

**Table 5:** Tabulated optical properties (absorbance and fluorescence) for four new Aza-Biotin conjugate fluorophores [66].

Sensitizer	λ_abs_ nm (ε, M^−1^ cm^−1^)	λ_em_ nm	Fluorescence QY, Φ_f_	Triplet QY Φ_T_	Singlet Oxygen Generation QY Φ (^1^O_2_)
DPR1a [66]	660 (106,500)	698	0.200	ND	ND
DPR1b [66]	684 (80,000)	721	0.300	ND	ND
DPR2a [66]	648 (67,000)	703	0.027	0.75	0.72
DPR2b [66]	670 (63,500)	706	0.065	0.79	0.75
Photofrin [66]	628 (3000)	630	0.10	0.61	0.30
Aza-BODIPY 5a [66]	660 (83,000)	706	-------	0.68	0.65

**Table 6:** UV-Vis and fluorescence spectroscopic properties of compounds **87**, **88**, **91**, and **92** with absorption and emission wavelength maximums, Stokes shift, and maximum molar absorptivity values displayed [74].

Compound	87	88	91	92
Solvent	DMF	DMF	DMF	DMF
λabs [nm] [74]	654	667	670	670
λem [nm] [74]	676	693	701	701
Δ λ [nm] [74]	22	26	31	31
εmax [M^−1^ cm^−1^] [74]	81,987	74,435	64,505	73,825
(±) (SD) [74]	(±7394)	(±4023)	(±5206)	(±5586)

**Table 7:** Experimental and M06-2X/6-31 G(d,p) calculated spectroscopic properties of Aza-BODIPYs in CHCl_3_ at room temperature [79]. Φf denotes the fluorescence quantum yield. ^[a]^ Aza-BODIPY 108 (Φf = 0.23 in CHCl_3_) were used as the standard for BODIPYs 108-113.

Compound	λabs [nm] Exp	λem [nm] Calc	Band Gap (eV)	ε [M^−1^ cm^−1^]	Oscillator Strength	λem [nm]	Φf ^[a]^	Stokes Shift [nm]
**108** [79]	664	594	3.70	78,000	0.90	691	0.23	27
**109** [79]	664	612	3.64	53,300	0.79	697	0.32	33
**111** [79]	642	572	3.84	33,800	0.74	696	0.13	54
**110** [79]	667	593	3.72	47,300	0.74	703	0.23	36
**115** [79]	653	576	3.83	72,800	0.91	692	<0.01	39
**112** [79]	654	580	3.82	41,100	0.79	693	<0.01	39
**114** [79]	644	569	3.88	36,100	0.80	696	<0.01	52
**113** [79]	648	584	3.81	53,200	0.74	699	<0.01	51

## Data Availability

No new data were created or analyzed in this study. Data sharing is not applicable to this article.

## Abbreviations

AC_2_O	Acetic Anhydride
a-TOH	a-Tocopherol
AIE	Aggregation-Induced Emission
aPS	Activatable photosensitizers
Aza-BDPBA	Aza-Boronic Acid Functionalized
BODIPY	Boron-dipyrromethene
BuOH	Butanol
CT	Charge Transfer
CuAAC	Cu(I)-catalyzed azide-alkyne Cycloaddition
Cys	Cysteine
DABCO	Triazabicyclo [2.2.2] octane
DCFH-DA	2′,7′-Dichlorofluorescin Diacetate
DFT	Density-Functional Theory
DDQ	(2,3-Dichloro-5,6-dicyano-1,4-benzoquinone)
DPBF	1,3-Diphenylisobenzofuran
DPP	Diketopyrrolopyrrole
ECL	Electrochemiluminescence
EDG	Electron Donating Group
EWG	Electron Withdrawing Group
FE	Fluorescent Enhancement
FRET	Förster Resonance Energy Transfer
GSH	Glutathione
ISC	Intersystem Crossing
HOMO	Highest Unoccupied Molecular Orbital
Hcy	Homocysteine
ICT	Intramolecular Charge Transfer
LCMS	Laser Confocal Microscopy Scanning
LOD	Limit of Detection
LOQ	Limit of Quantification
LUMO	Lowest Unoccupied Molecular Orbital
MO	Molecular Orbital
MCR	Multicomponent Reactions
NAC	*N-*acetylcysteine
NIR	Near-Infrared Region
NIR-II	Near-Infrared Region Window II
NP	Nanoparticles
OL	Olive Oil
PA	Picric Acid
PAI	Photoacoustic Imaging
PALM	Photoactivated Localization Microscopy
PBA	Phenyl Boronic Acid
PEG-3	Polyethylene Glycol
PDT	Photodynamic Therapy
PET	Positron Emission Tomography
PET	Photoinduced Electron Transfer
PM	Plasma Membrane
PPAB	Pyrrolopyrrole Aza-BODIPY
PS	Photosensitizers
PTPE_3_ NPs	Pyrrolopyrrole aza-BODIPY nanoparticles
RDX	Hexahydro-1,3,5-trinitro-1,3,5-triazine
RNS	Reactive Nitrogen Species
ROSs	Reactive Oxygen Species
RNSs	Reactive Nitrogen Species
SBR	Signal-to-Background Ratio
SNR	Signal-to-Noise Ratio
THF	Tetrohydrofuran
TPP	Triphenylphosphonium
TPE	Tetraphenylethylene
TOH	Tocopherol
V-PDT	Vascular Photodynamic Therapy
UV	Ultraviolet
